# Integration of genome-level data to allow identification of subtype-specific vulnerability genes as novel therapeutic targets

**DOI:** 10.1038/s41388-021-01923-1

**Published:** 2021-07-06

**Authors:** Edward C. Schwalbe, Lalchungnunga H, Fadhel Lafta, Timothy M. Barrow, Gordon Strathdee

**Affiliations:** 1 Biosciences Institute, Newcastle University Centre for Cancer, Newcastle University, Newcastle, UK; 2 Department of Applied Sciences, Northumbria University, Newcastle, UK; 3 Department of Biology, College of Science, University of Baghdad, Baghdad, Iraq; 4 Faculty of Health Sciences and Wellbeing, University of Sunderland, Sunderland, UK

**Keywords:** Cancer genomics, CNS cancer, Acute lymphocytic leukaemia, Target identification, Epigenomics

## Abstract

The identification of cancer-specific vulnerability genes is one of the most promising approaches for developing more effective and less toxic cancer treatments. Cancer genomes exhibit thousands of changes in DNA methylation and gene expression, with the vast majority likely to be passenger changes. We hypothesised that, through integration of genome-wide DNA methylation/expression data, we could exploit this inherent variability to identify cancer subtype-specific vulnerability genes that would represent novel therapeutic targets that could allow cancer-specific cell killing. We developed a bioinformatics pipeline integrating genome-wide DNA methylation/gene expression data to identify candidate subtype-specific vulnerability partner genes for the genetic drivers of individual genetic/molecular subtypes. Using acute lymphoblastic leukaemia as an initial model, 21 candidate subtype-specific vulnerability genes were identified across the five common genetic subtypes, with at least one per subtype. To confirm the approach was applicable across cancer types, we also assessed medulloblastoma, identifying 15 candidate subtype-specific vulnerability genes across three of four established subtypes. Almost all identified genes had not previously been implicated in these diseases. Functional analysis of seven candidate subtype-specific vulnerability genes across the two tumour types confirmed that siRNA-mediated knockdown induced significant inhibition of proliferation/induction of apoptosis, which was specific to the cancer subtype in which the gene was predicted to be specifically lethal. Thus, we present a novel approach that integrates genome-wide DNA methylation/expression data to identify cancer subtype-specific vulnerability genes as novel therapeutic targets. We demonstrate this approach is applicable to multiple cancer types and identifies true functional subtype-specific vulnerability genes with high efficiency.

## Introduction

Cancer is widely recognised as a genetic and epigenetic disease [1]. Large-scale changes in the genetic and epigenetic landscape in cancer cells lead to cellular transformation [2]. Alterations in epigenetic mechanisms, particularly alterations in DNA methylation, have been found in all cancer types. It is now clear that inactivation due to promoter methylation is one of the primary mechanisms leading to the loss of expression of tumour suppressor genes, such as *MLH1* and *BRCA1* [3].

Genome-wide approaches have revealed the large extent of altered DNA methylation in cancers, where several thousand promoter-associated CpG islands become hypermethylated in a single tumour [4]. Moreover, it has become further apparent that methylation changes are not restricted to gene promoters and that the overall patterns of altered DNA methylation in cancers are highly complex [5].

DNA methylation profiling approaches have determined that each cancer type is associated with a characteristic DNA methylation “fingerprint” [6, 7]. Within specific tumour types, methylation profiling can be used to identify specific subtypes, often associated with differences in clinical behaviour and outcomes. In childhood acute lymphoblastic leukaemia (ALL), it has been shown that methylation profiles are strongly linked to cytogenetic differences, such that clustering of methylation data can recapitulate the same genetic subtypes achieved through cytogenetic analysis [8, 9]. In multiple other types of cancer, methylation profiling can identify biologically distinct subgroups with distinct biological and clinical characteristics [10]. Thus, within a specific tumour type, there are a set of methylation changes that are shared across all/most patients, and a second set of methylation alterations that are subtype-specific.

While classifying cancer has become easier due to the ability to assay large numbers of CpG loci, the scale of the changes makes it very challenging to distinguish the presumably small number of biologically crucial changes from the very large number of passenger events. Similarly, gene expression differences between subtypes also typically involve very large numbers of genes [11], making it equally challenging to identify key expression changes. However, here we present an approach that can actively exploit the large number of passenger changes in DNA methylation and gene expression and uses this to identify subtype-specific vulnerability (SSV) partner genes for the genetic changes underlying the development of specific genetic/molecular subtypes of cancer. In essence, this approach, through the integration of genomic methylation and expression data, identifies genes that are prevented from acquiring passenger methylation (and whose expression is retained), only in the presence of a specific cancer-causing mutation/pathway. This implies selection for retention of expression of the gene only in presence of that specific cancer-driving mutation. Identification of such SSV genes will be crucial for improving cancer therapy, as they are ideal targets for the development of novel therapeutic strategies, which can specifically target cancer cells and limit or prevent normal cell toxicity [12].

Using ALL and medulloblastoma as models, we demonstrate that our approach can identify candidate SSV genes in almost all commonly recognised genetic/molecular subtypes of these cancers. Furthermore, modulation of expression of candidate SSV genes in ALL/medulloblastoma cell lines confirms that loss of expression was specifically lethal in the predicted molecular subtypes for all tested candidates (*n* = 7). This approach is likely to be widely applicable for the identification of SSV genes across all/many cancer types that could facilitate the development of therapeutic approaches which can specifically target cancer cells, while having limited or no toxicity against normal cells.

## Results

### Most identified methylation changes are shared across all ALL genetic subtypes, also occur in normal proliferating B-cells and are independent of disease or transformation

An important step in the development of our approach for identifying cancer subtype-specific SSV genes, is a clearer understanding of the underlying forces that drive the extensive changes in genome-wide methylation and how this relates to disease development. It has recently been shown in B-cell malignancies that many hypermethylation events that were thought to be disease-specific also occur in the latter stages of B-cell development [13, 14]. To confirm this was similarly true in ALL subtypes, we assessed a list of 9,348 CpG sites previously identified as altered in all ALL genetic subtypes (compared with CD19-positive B lymphocytes) [9]. 8760/9348 (93.7%) of the CpG sites exhibit altered DNA methylation in the same direction seen in ALL when terminally differentiated class-switched memory B-cells were compared to naïve B-cells (*p* = 8 × 10^−64^, Table 1). Over 80% of these sites exhibit beta changes of >0.1 (equivalent to an approximately >10% change in absolute methylation) when comparing class-switched memory B-cells to naïve B-cells. Of these larger changes, 7371/7518 (98%) are altered in the same direction in class-switched memory B-cells as in ALL cells (*p* = 2 × 10^−68^, Table 1B).

**Table 1:** Comparison of methylation changes in ALL and normal B cells.

		Methylation change in ALL^a^	
Methylation change in normal CS memory B cells^b^		Hypermethylated	Hypomethylated
	Hypermethylated	8680	45
	Hypomethylated	543	80

		Methylation change in ALL	
Methylation change (>10%) in normal CS memory B cells^c^		Hypermethylated	Hypomethylated
	Hypermethylated	7321	20
	Hypomethylated	127	50

^**a**^9348 CpG sites identified by Nordlund et al.^9^ as exhibiting altered methylation (beta value change of >0.2) in all ALL genetic subtypes (compared with total CD19-positive cells).

^b^Changes in the same direction as in ALL were observed in CS (class switched) Memory B cells (compared with naïve B cells) for 93.7% of CpG sites (*p* = 8 × 10^−64^, Fisher Exact Test).

^c^Analysis was restricted to CpG sites exhibiting large changes (beta value change of >0.1) in CS memory cells (found in 7518/9348 CpG sites (80.4%)). Sites with such large changes were even more likely to mirror changes seen in ALL ((98% of CpG sites change in the same direction, *p* = 2 × 10^−68^, Fisher Exact Test).

This suggests that the overwhelming majority of methylation changes seen in ALL are not disease-specific and can occur in B lymphocytes regardless of transformation status. Crucially, this implies that there are a large number of CpG sites that invariably acquire altered methylation in all clones of proliferating B-cells, whether they are transformed or not, unless there is a specific selective pressure to prevent it. Based on this, we hypothesised that if these “inevitable” methylation changes happened to occur at a gene that was a SSV partner for a specific cancer-causing mutation, then this SSV gene partner of the cancer-causing mutation could be identified by the retention of low methylation (and continued gene expression) only within one specific genetic/molecular subtype. A further advantage of any such identified genes is that, as they are highly methylated and unexpressed in all other subtypes of that cancer, we know, a priori, that the gene cannot be generally required for normal cell survival/proliferation.

### Outline of approach for integration of genome-wide DNA methylation and gene expression data to identify subtype-specific SSV genes

**Fig. 1:**
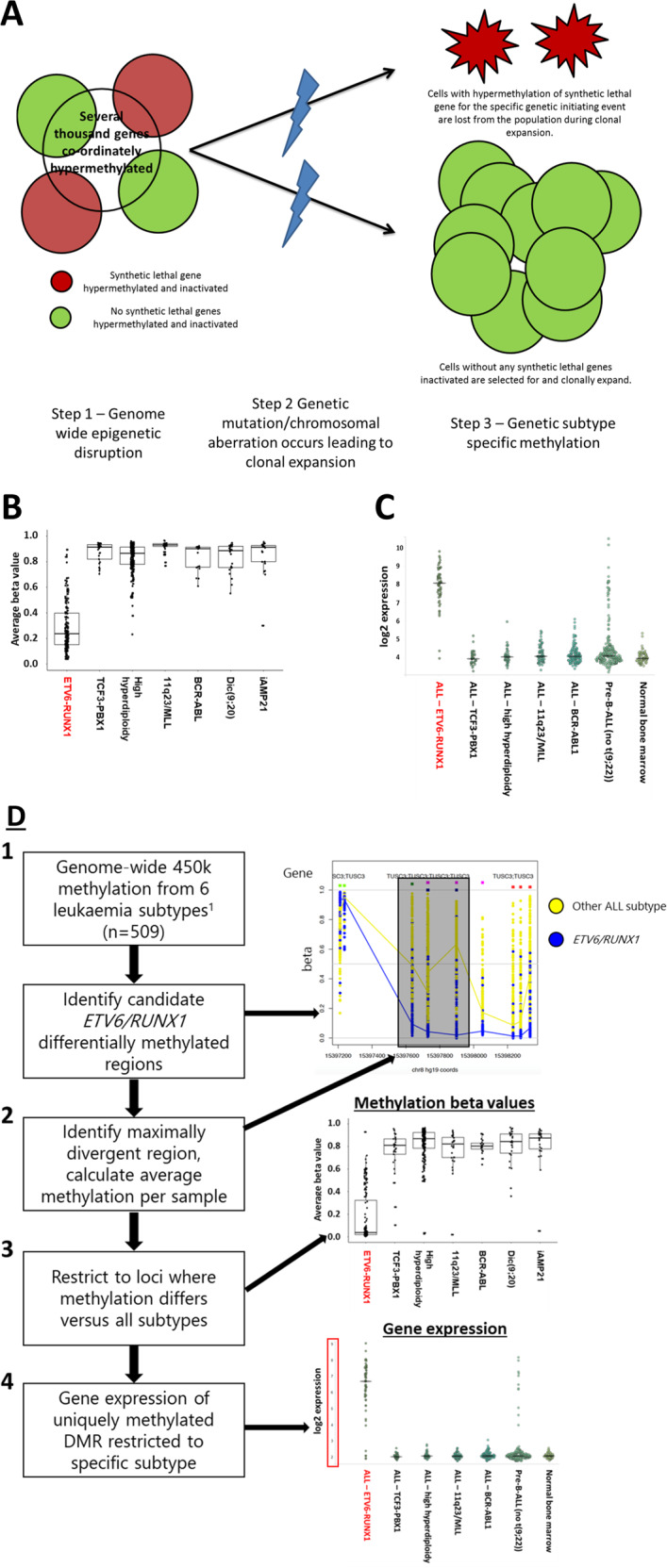
Outline of the process that leads to the proposed generation of SSV genes in ALL. **A** Step 1—Co-ordinated methylation of thousands of promoter-associated CpG islands—seen in essentially all ALL cases. This will result in a mixed population of cells some with (red cells) and some without (green cells) inactivation of specific SSV genes. Step 2—Initiating genetic lesion drives clonal expansion of epigenetically disrupted cells. Step 3—The result is a natural “experiment” in which all genes affected at step 1 are “tested” for subtype-specific lethality. i.e. cells in which a SSV gene for the specific genetic initiating mutation is methylated and inactivated will be lost during clonal expansion (red cells), while cells with no SSV genes methylated/inactivated will clonally expand. Subsequently we can identify SSV genes based on their unusual methylation patterns (i.e. high methylation in all other subtypes, but very low methylation in the specific genetic subtype for which the gene is specifically lethal). For illustrative purposes genome-wide and subtype-specific methylation changes (driven by the initiating genetic event) are shown as occurring sequentially. They may occur simultaneously, during the initial stages of clonal expansion driven by the subtype-specific defining mutation, but this would not alter the hypothesis as hypermethylation of SSV genes would still be selected against only in the genetic subtype in which they are specifically lethal. **B** Illustrative example of the methylation differences identified between genetic subtypes for potential SSV genes. These illustrative examples show the methylation difference between ETV6-RUNX1 ALL and all other subtypes at a DMR overlapping the *DSC3* promoter region. This is typical of the predicted pattern of methylation for SSV genes derived as described in (**A**). **C** Integration with gene expression data. Only genes where the altered DNA methylation is associated with the predicted subtype-specific gene expression are retained as candidate SSV genes. In this example, the candidate gene (*DSC3*) exhibits high expression in the subtype of interest (B-ALL *ETV6-RUNX1*) but low/absent expression in all other subtypes, thus demonstrating that it is not generally required for cell growth/survival for B-cells. This suggests that targeting such a gene should specifically affect the cancer cells with the particular cancer-initiating mutation. **D** Flow diagram of the bioinformatics pipeline for identification of SSV gene candidates. (1) All DMRs for a specific subtype of cancer under study are initially selected using DMRcate. (2) The initial region is then further analysed to identify the maximally divergent region (must contain at least 2 CpG sites). (3) This region is then tested in comparison with all other subtypes of cancer under study and only regions that are divergent from every other subtypes are retained as markers of potential SSV genes. (4) Gene expression data sets are used to analyse the expression of the nearest gene from loci derived from step 3, to identify those in which the reduced methylation is associated with subtype-specific gene expression. Genes in which expression is exclusive to the subtype of interest are taken forward as SSV candidates.

Genome-wide DNA methylation changes in ALL can be broadly separated into changes shared across all ALL subtypes, as described above, and subtype-specific changes [9]. This altered DNA methylation can be viewed as occurring in two different processes—one that affects a very large number of loci that are shared across all subtypes, and is likely a result of extensive proliferation. Then the second, affecting a smaller number of loci, modulates this pattern according to the biological differences induced by different genetic initiating events (Fig. 1A). As illustrated in Fig. 1A, it is this second phase in which differential patterns of DNA methylation/gene expression might be selected for at SSV partner genes for the specific mutation driving that cancer. This is because, from the large number of genes affected in the proliferation-dependent wave, any gene whose expression is required for the growth/survival of cells bearing that specific genetic initiating mutation will be strongly selected to retain low methylation (if that methylation negatively impacts gene expression, see integration with gene expression data below). This will result in low methylation in that specific genetic subtype but high methylation in all other subtypes (example in Fig. 1B). This is in contrast to genes required more generally for survival of progenitor B-cells, which would exhibit low methylation in all ALL subtypes and normal cells. Importantly, methylation acts *in cis* and thus the selected methylation difference can only impact the expression of the linked gene, allowing the specific target of this selective process to be identified.

Genome-wide methylation analysis is then integrated with genome-wide expression data, to restrict the further analysis to loci at which the altered DNA methylation is associated with a corresponding change in gene expression (Fig. 1). In addition to positive expression of the candidate gene in the subtype of interest, SSV genes should exhibit very low/absent expression in all other subtypes and in normal haematopoietic cells. This would thereby demonstrate that the gene is not required for the growth/survival of B-cells in general (example in Fig. 1C). By combining the methylation and expression data in our candidate selection, this ensures that selection is occurring directly at that specific locus (as methylation only acts *in cis*) and that it leads to a biological effect (as expression of the candidate is restricted to the subtype being studied) and that we identify only candidates that are not required for normal B cell growth/survival (as expression is low/absent in all other subtypes and normal cells).

#### Identification of SSV genes in specific genetic subtypes of ALL

As proof of principle of the approach described above, we initially studied the two most common ALL genetic subtypes driven by single specific genetic events; the *ETV6-RUNX1* and *TCF3-PBX1* subtypes of ALL. A bioinformatic pipeline was designed to enable this analysis (outlined in Fig. 1D). Our own [8] and publicly available genome-wide DNA methylation analysis [9] was integrated with a publicly available gene expression data set (Leukaemia Mile Study [15]) to enable the identification of SSV gene candidates.

**Fig. 2:**
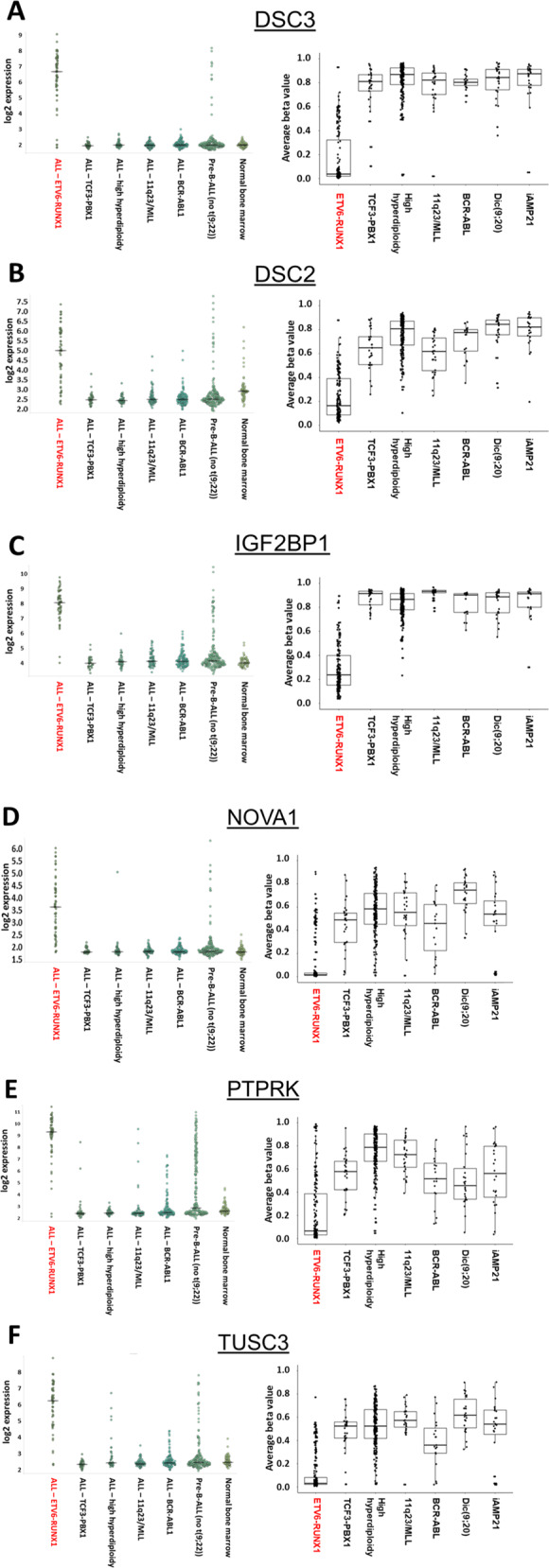
Methylation and gene expression patterns of all SSV genes identified in the ETV6/RUNX1 subtype. Gene expression plots in B-cell ALL genetic subtypes (derived from BloodSpot, http://servers.binf.ku.dk/bloodspot/) and DNA methylation data from the region of largest change within each DMR are shown for all six candidate SSV genes identified in the ETV6-RUNX1 subtype.

We identified six candidate genes that fulfilled all criteria in *ETV6-RUNX1* and nine in *TCF3-PBX1* (details listed in Table 2, methylation and expression levels for *ETV6-RUNX1* are illustrated in Fig. 2, with differentially methylated regions (DMRs) shown in their genomic context in supplementary Fig. 1, methylation and expression levels for *TCF3-PBX1* are illustrated in supplementary Fig. 2). Most of the identified genes have not been studied for their role in ALL, emphasising that this approach is likely to generate novel potential target genes, with the exception of *IGF2BP1* (*ETV6-RUNX1*) and *WNT16* (*TCF3-PBX1*). *IGF2BP1*, has previously been identified as overexpressed in *ETV6-RUNX1* ALL. It is required for stability of the *ETV6/RUNX1* fusion transcript [16] and *IGF2BP1* knockdown inhibits growth and survival of *ETV6-RUNX1* positive cells [17], compatible with a SSV function in *ETV6-RUNX1* positive ALL. Similarly, *WNT16* has previously been identified as overexpressed in *TCF3-PBX1* positive ALL and is required for cell survival of *TCF3-PBX1* positive cells [18].

**Table 2:** Candidate SSV genes identified in ALL genetic subtypes.

ETV6-RUNX1						
Gene symbol	EntrezID	Chr	Distance to TSS	DMR_Start^a^	DMR_End	Size of DMR
*DSC3*	1825	chr18	0	28620915	28624074	3159
*DSC2*	1824	chr18	0	28681121	28683794	2673
*IGF2BP1*	10642	chr17	15177	47089952	47092272	2320
*NOVA1*	4857	chr14	0	27065974	27069329	3355
*PTPRK*	5796	chr6	0	128839906	1.29E + 08	2499
*TUSC3*	7991	chr8	0	15397211	15398333	1122

TCF3-PBX1						
Gene symbol	EntrezID	Chr	Distance to TSS	DMR_Start	DMR_End	Size of DMR
*CHST6*	4166	chr16	0	75528459	75529892	1433
*DCHS2*	54798	chr4	0	155410477	155413789	3312
*FAT1*	2195	chr4	0	187644620	187648358	3738
*NLGN1*	22871	chr3	0	173113110	173116248	3138
*PHACTR3*	116154	chr20	0	58151913	58152803	890
*SPAG6*	9576	chr10	0	22633916	22635028	1112
*TCERG1L*	256536	chr10	0	133109013	133111331	2318
*UGT8*	7368	chr4	0	115518992	115521241	2249
*WNT16*	51384	chr7	0	120967900	120970606	2706

High hyperdiploidy						
Gene symbol	EntrezID	Chr	Distance to TSS	DMR_Start	DMR_End	Size of DMR
*CADPS*	8618	chr3	0	62859289	62861925	2636
*PLVAP*	83483	chr19	0	17487776	17489311	1535

MLL/11q23						
Gene symbol	EntrezID	Chr	Distance to TSS	DMR_Start	DMR_End	Size of DMR
*SKIDA1*	387640	chr10	1446	21802824	21805402	2578
*ZC3H12C*	85463	chr11	0	109962727	109964976	2249

BCR/ABL1						
Gene symbol	EntrezID	Chr	Distance to TSS	DMR_Start	DMR_End	Size of DMR
*MIRLET7BHG*	400931	chr22	4750	46468422	46471442	3020
*PDK4*	5166	chr7	0	95225520	95226433	913

^a^Based on the GRCh37 genome assembly.

Our hypothesis further predicts that methylation patterns of the identified SSV genes should be reflective of naive B-cells in the subtype in which they are specifically lethal (i.e. will have retained the methylation patterns seen in normal cells that have not undergone extensive proliferation). In contrast, methylation patterns in all other ALL subtypes should mirror that seen in class-switched memory B-cells (i.e. normal cells that have undergone extensive proliferation). Analysis of individual CpG sites across the six *ETV6-RUNX1* candidates (Supplementary Table 1), confirmed that the methylation patterns across these genes match that seen in the predicted normal cell populations. This emphasises that the methylation patterns at the identified SSV gene candidates are reflective of normal states. The disease-specific event is not the hypermethylation of these genes, but the retention of the non-proliferative/naive cell pattern in the genetic subtype in which the gene was identified as a SSV candidate.

**Fig. 3:**
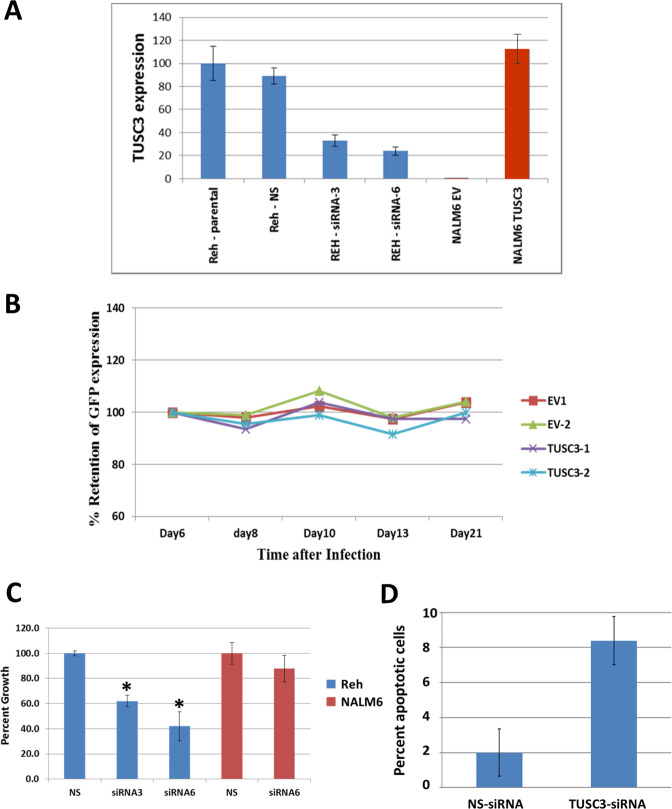
*TUSC3* expression is required for the growth and survival of *ETV6-RUNX1* expressing cells. **A***TUSC3* expression as determined by qRT-PCR in Reh cells with and without treatment with siRNA directed against *TUSC3* and also in NALM6 cells before and after transduction with a *TUSC3* expression construct. **B** *TUSC3* expression has no significant impact on NALM6 cells. NALM6 cells were transduced with an empty vector, expressing GFP alone (EV) or a *TUSC3* expression vector, also expressing GFP (*TUSC3*). The retention of GFP-expressing cells was followed in a mixed population. As expected expression of GFP alone had no impact on growth survival, as illustrated by the fraction of GFP-expressing cells remaining essentially constant over 21 days. Similarly, *TUSC3* expressing cells also remained constant over the assessment period, indicating that modulation of *TUSC3* expression had no significant impact on the growth of *ETV6-RUNX1* negative NALM6 cells. **C** Knockdown of *TUSC3* in Reh cells significantly reduces cell growth. Knockdown of *TUSC3* with two different siRNA constructs resulted in a 65–75% reduction in expression (see (A)). Cell growth by 4 days following knockdown with either construct was very significantly reduced, as compared with control non-silencing RNA (NS-siRNA). *p < 0.01. **D** Knockdown of *TUSC3* induces apoptosis in Reh cells. Forty-eight-hours post transfection, apoptosis was assessed in Reh cells exposed to *TUSC3* specific siRNA versus control non-silencing siRNA. While treatment with the control siRNA resulted only in a very low level of apoptosis, levels of apoptosis were significantly higher following treatment with *TUSC3* specific siRNA (*p* < 0.01). Overall these results show that modulation of *TUSC3* expression levels specifically inhibits cell growth/survival in *ETV6-RUNX1* positive cells.

We selected one candidate from each genetic subtype (*TUSC3* for *ETV6-RUNX1* and *FAT1* for *TCF3-PBX1*) for function assessment. For *TUSC3*, this analysis utilised the *ETV6-RUNX1* positive REH cell line (which expresses *TUSC3*, as predicted for ETV6-RUNX1 positive ALL cells) and the *ETV6-RUNX1* negative NALM6 cell line (in which *TUSC3* is hypermethylated and inactivated, as predicted for ETV6-RUNX1 negative ALL cells) (Fig. 3A). As *TUSC3* has also previously been suggested as a potential tumour suppressor gene, in part due to its frequent hypermethylation [19], we also assessed any negative impact on the growth of re-expression of the gene in the TUSC3-deficient NALM6 cell line. In REH cells, expression of *TUSC3* was reduced (65–75%) by treating with either of two different siRNA constructs targeted against *TUSC3* (Fig. 3A). Although knockdown was only partial, this had a significant impact on cell growth, resulting in 40–60% reduction in cell numbers 4 days post treatment (Fig. 3C). Furthermore, knockdown of *TUSC3* resulted in induction of apoptosis specifically in the *ETV6-RUNX1* positive REH cells (Fig. 3D). As expected, treatment of *TUSC3* negative NALM6 cells with *TUSC3* siRNA had no impact on cell growth or apoptosis (Fig. 3C, D). Furthermore, re-expression of *TUSC3* in NALM6 cells, at levels equivalent to the endogenous expression in REH (Fig. 3A), had no detectable impact on the growth of the NALM6 cells (Fig. 3B), indicating that these cells are insensitive to modulation of *TUSC3* expression level.

**Fig. 4:**
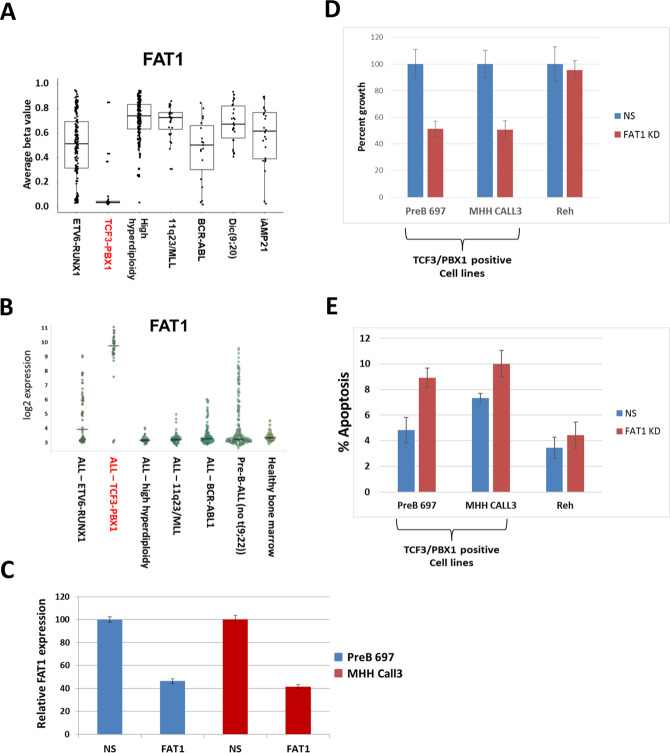
Knockdown of *FAT1* specifically alters the growth/survival of *TFC3-PBX1* cells. *FAT1* exhibits *TCF3-PBX1* specific patterns of (**A**) Methylation and (**B**) expression, compatible with TCF3-PBX1 subtype-specific lethality. **C** Treatment of two different *TCF3-PBX1* positive cell lines (PreB 697 and MHH cALL3) with a siRNA against *FAT1* results in an ~60% reduction in *FAT1* expression 48-hours post transfection, as assessed by qRT-PCR. **D** Cell growth following knockdown with *FAT1* specific siRNA is dramatically reduced compared with non-silencing siRNA in both *TCF3-PBX1* positive cell lines (*p* < 0.002 for both). In contrast no impact is seen in the *TCF3-PBX1*/*FAT1*-negative cell line Reh, indicating that this is not due to an off-target effect. **E** Treatment with *FAT1* siRNA results in a small but statistically significant increase in apoptosis in *TCF3-PBX1* positive cell lines, as compared to control non-silencing siRNA (*p* < 0.05 for both). As expected, *FAT1* siRNA has no significant impact on apoptosis levels in FAT1-negative Reh cells.

Similarly, *FAT1* function was assessed in *TCF3-PBX1* positive ALL cell lines (PreB697 and MHH CALL3), which express FAT1 and exhibit low *FAT1* methylation (as seen in *TCF3-PBX1* primary samples), as well as a control *TCF3-PBX1*-negative line REH, in which the *FAT1* gene is hypermethylated and not expressed. As seen for *TUSC3* in the *ETV6-RUNX1* subtype, partial siRNA-mediated knockdown of *FAT1* resulted in inhibition of cell growth and induction of apoptosis in cell lines from the relevant subtype (i.e. PreB697 and MHH CALL3). In contrast, it had no impact on cells from a different genetic subtype (i.e. Reh) (Fig. 4). Thus, for both genes, retention of expression was required for cell growth and survival, exclusively in cells bearing the cancer-causing mutation that the genes were predicted to be specifically lethal with.

#### Identification of SSV candidates in other ALL subtypes

To investigate the wider applicability of this approach, we expanded this initial analysis to other ALL subtypes, including those with less specific genetic changes (e.g. high hyperdiploidy). Using our approach, SSV gene candidates were identified in all genetic subgroups in ALL (Table 2; DNA methylation and gene expression data for all identified candidates is detailed in supplementary Fig. 2). As for the above analysis, most identified genes have not been widely studied for their role in leukaemia and represent potentially novel therapeutic targets. Thus the approach described here can be used to identify SSV gene candidates across subtypes defined by a wide range of known and less defined molecular abnormalities.

#### Identification of SSV gene candidates in medulloblastoma

After proof of principle across ALL subtypes, we next investigated whether SSV gene candidates could be identified in a non-haematological cancer, medulloblastoma. This CNS tumour comprises four molecularly defined subgroups (WNT, SHH, Group 3 and Group 4), defined through international consensus and which are identifiable by transcriptomic or methylomic signatures [20, 21]. Using our novel approach, SSV gene candidates were identified in WNT, SHH and Group 4 subgroups (Table 3, DNA methylation and gene expression data for all identified candidates is detailed in supplementary Fig. 3). Again, identified genes represent potentially novel therapeutic targets. Furthermore, the medulloblastoma dataset included both adult and childhood patients and the identification of SSV gene candidates was found to be dependent on the molecular subgroup but independent of patient age (Supplementary Fig. 3). These results suggest that the reported approach could have broad applicability across different cancer types.

**Table 3:** Candidate SSV genes identified in medulloblastoma subtypes.

WNT						
Gene symbol	EntrezID	Chr	Distance to TSS	DMR_Start^a^	DMR_End	Size of DMR
*LINC01124*	440925	chr2	0	171568412	171574592	6180
*LRP4*	4038	chr11	0	46939436	46941490	2054
*MIR193A*	406968	chr17	0	29885787	29888936	3149
*NKD1*	85407	chr16	0	50580632	50585731	5099
*PHETA2*	150368	chr22	0	42469756	42470868	1112
*RASIP1*	54922	chr19	0	49242037	49244965	2928
*SYK*	6850	chr9	0	93563776	93564603	827

SHH						
Gene symbol	EntrezID	Chr	Distance to TSS	DMR_Start	DMR_End	Size of DMR
*ATOH1*	474	chr4	5441	94755520	94756895	1375
*CPLX1*	10815	chr4	7060	811641	812884	1243
*CRIP2*	1397	chr14	5328	105944604	105946891	2287
*FOXS1*	2307	chr20	0	30431758	30434529	2771
*GPR68*	8111	chr14	18919	91699881	91701304	1423
*PNPLA2*	57104	chr11	3495	822397	824970	2573
*SPHK1*	8877	chr17	8471	74381214	74383906	2692

Group 3						
Gene symbol	EntrezID	Chr	Distance to TSS	DMR_Start	DMR_End	Size of DMR
None identified						

Group 4						
Gene symbol	EntrezID	Chr	Distance to TSS	DMR_Start	DMR_End	Size of DMR
*LMX1A*	4009	chr1	0	165321224	165327830	6606

^a^Based on the GRCh37 genome assembly.

### Functional assessment of SSV gene candidates in medulloblastoma

**Fig. 5:**
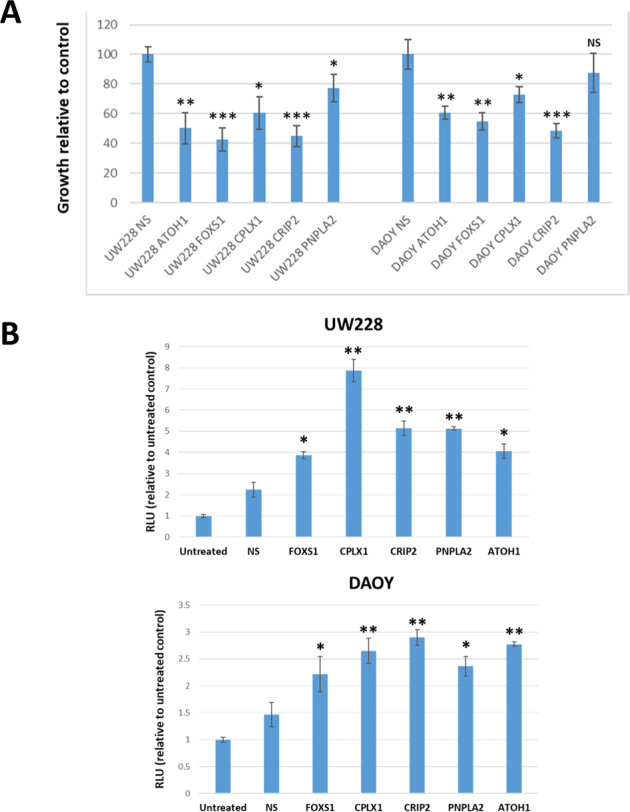
siRNA-mediated knockdown of candidate SHH SSV genes reduces proliferation and induces apoptosis in SHH group medulloblastoma cell lines. **A** Two SHH-derived MB cell lines (UW228, DAOY) were transfected with non-silencing control siRNA or siRNA against five different SSV gene candidates, predicted to be specifically lethal in the SHH subtype. Cells were assayed by MTT assay 3 days post transfection (*n* = 6). *p < 0.05, **p < 0.005, ***p < 0.001, NS = not statistically significant. **B** 48 h post-transfection cells were assessed for apoptosis by measuring caspase activation (*n* = 3–6). Significantly increased caspase activation was detected following transfection with all five of the gene-specific siRNAs in both cell lines. *p < 0.05, **p < 0.005. The results indicate that for all five candidate SSV genes, loss of expression was associated with reduced cell survival of SHH medulloblastoma cells.

Most of the identified SSV gene candidates were in the WNT and SHH subtypes. However, as cell line models are not available for WNT medulloblastoma, we focused on functional validation of the SHH candidates. siRNA were obtained for five of the SHH candidate SSV candidates and used to transfect two SHH medulloblastoma cell lines (UW228 and DAOY). Knockdown of each of the five candidate genes induced a significant reduction in cell numbers in the SHH medulloblastoma cell lines, demonstrating that targeting these genes was able to induce anti-proliferative effects in SHH medulloblastoma cells as predicted (Fig. 5A). The relative impact of targeting the genes was variable. Knockdown of the *PNPLA2* gene only resulted in a minor reduction of about 20% (which was only statistically significant in one of the two cell lines), while knockdown of the majority of the candidates resulted in larger decreases of 40–60% relative to the non-silencing control (Fig. 5A). Furthermore, we examined caspase activation, as a measure of apoptosis. Again, knockdown of all five candidate genes resulted in significant induction of caspase activity, implying that knockdown of the candidate genes was at least in part cytotoxic (Fig. 5B). Assessment of the efficiency of knockdown at the RNA level by qRT-PCR identified reduced expression of the targeted genes 48 h post siRNA transfection (supplementary Fig. 4). Protein expression was also assessed for three of the targets (*ATOH1*, *CRIP2* and *PNPLA2*), demonstrating protein levels were also reduced at 48 h post-transfection (supplementary Fig. 4B). Thus, all five tested genes exhibited functional activity consistent with subtype-specific lethality, suggesting that the approach identifies functional SSV genes with a high degree of efficiency.

## Discussion

The large extent and complexity of molecular changes occurring in cancer makes the identification of key changes very challenging. Here we present a novel approach, integrating genome-wide DNA methylation and expression data, that can actually utilise the very large number of passenger changes to allow identification of candidate SSV genes. We demonstrate this approach can identify SSV gene candidates in almost all molecular subtypes assessed and functional analysis validated all candidate genes tested. siRNA-mediated knockdown of all seven of the tested candidates resulted in reduced cell growth and the induction of apoptosis, suggesting that the approach we report here identifies SSV genes with a high degree of efficiency. We further demonstrated that SSV genes could be identified in both adult and childhood medulloblastoma. As similar extensive DNA methylation changes occur in all tumour types it is likely that this approach will be applicable to many other cancers. This could allow the identification of novel therapeutic targets for the development of therapies with the potential to specifically target cancer cells, while achieving minimal normal cell toxicity. This would be crucial in improving cancer outcomes, enabling treatment in less robust patient groups, such as the elderly, and in reducing the long-term health effects suffered by survivors of childhood/young adult cancer.

This study focussed on the identification of SSV genes that are required in specific molecular subtypes of cancer. While most cancers were initially classified based on morphology, detailed molecular analysis has suggested the different molecular subtypes are quite distinct, displaying differences in patient outcome [22], gene expression profiles [23] and genome-wide methylation [9]. Consequently, each molecular subtype is likely to exhibit its own specific SSV genes and co-operating mutations. Consistent with the existence of molecular subtype-specific lethal genes, we found unique candidates in almost all molecular subtypes analysed in ALL and medulloblastoma. However, it is also possible that SSV genes may be shared across some or all different genetic subtypes and modifications of our current approach will be utilised to assess this in subsequent studies.

A key to our approach for identifying such subtype-specific lethal genes, lies in the unique features of abnormal methylation present in all cancer types. The comparatively high error rate in replicating methylation patterns results in the accumulation of high levels of aberrant promoter-associated methylation, which is incompatible with expression of the linked gene. As many genes are either not expressed or not required for growth/survival in specific cell types, the genes impacted by such aberrant methylation in a specific cell type are quite consistent and therefore predictable. However, as illustrated in Fig. 1, if a gene is a SSV gene in one specific molecular subtype, there will be a very strong selective pressure, only in this subtype, to maintain low methylation that is compatible with retained expression. Thus, genes required for survival specifically in one molecular subtype can be identified by a specific failure to accumulate methylation, which does accumulate in all other subtypes where the gene is not required. Importantly, methylation changes function in *cis* and thus alterations at a specific promoter will only affect the linked gene. Thus, the gene targeted by the differential methylation can readily be identified. Consistent with this hypothesis, all seven genes functionally assessed in this study exhibited subtype-specific lethal properties, suggesting that the approach we describe can identify functional SSV genes with a high degree of specificity.

Three genes identified by this approach in ALL and medulloblastoma have already been implicated with activity consistent with subtype-specific lethality. However, the majority of candidates identified have not previously been implicated in leukaemia/medulloblastoma biology. This includes six of the seven genes in which SSV gene activity was functionally confirmed. Key to this identification of previously unappreciated genes, is that the approach relies on the identification of genes that are not genetically disrupted and whose methylation patterns initially appear to reflect that seen in normal cells and thus would not be identified by standard epigenetic/genetic screening. Thus, the approach reported here could allow the identification of a large number of previously unidentified therapeutic targets across a wide range of cancers.

Whilst previous analysis of genetic/epigenetic changes would not have readily identified the genes identified here, some could have been identified solely through subtype-specific overexpression (as was the case for *IGF2BP1* [16, 17] and *WNT16* [18] in ALL and *ATOH1* [24] in medulloblastoma). However, the number of expression differences between subtypes is typically very high. Ross et al. [11] identified close to one thousand discriminating differences in expression in multiple ALL genetic subtypes. This is likely because altered expression of a specific gene usually reflects alterations to upstream regulators, such as transcription factors or signalling pathways. These act *in trans* and can alter the expression, directly and indirectly, at very large numbers of genes. Therefore, gene expression patterns alone cannot readily be used to identify changes that are of key functional importance. This is in contrast to DNA methylation changes, which, as discussed above, act in *cis*. Consequently, the inclusion of DNA methylation data greatly enhances the ability of our approach to identify genes under direct selection, as opposed to secondary passenger changes. The high “hit-rate” we obtained in the seven tested candidate SSV genes implies that this is a highly efficient method for identifying true functional SSV genes.

The majority of ALL patients have one of a number of well-defined genetic abnormalities and these abnormalities are thought to be critical initiating events for the disease [25]. Whilst the number of candidate SSV genes identified varied (from 2 to 9 candidates), potential SSV genes were identified for all genetic subtypes assessed. This implies that the approach we are using can identify candidate SSV genes across a broad range of different genetic initiating events. It may be that the approach will be most successful for subtypes with clearly defined molecular changes (i.e. *ETV6/RUNX1*, *TCF3-PBX1*, BCR-ABL1) as opposed to subtypes with more heterogeneous changes (i.e. HeH, MLL/11q23), as a greater number of candidate SSV genes were found for the former group than the latter. Similarly, in medulloblastoma, analysis of the well-defined *WNT* and *SHH* subgroups identified seven candidate SSV genes in each. In contrast, identification of SSV genes was limited in group 3/group 4 tumours, which lack a known subgroup-defining molecular defect and show evidence for overlapping biology [10, 26].

Several other techniques have been reported for identifying synthetic lethal or SSV genes, including approaches that utilise siRNA, shRNA and CRISPR libraries [27, 28] and bioinformatic approaches [29–31]. This has led to the development of an extensive database of potential synthetic lethal interactions [32]. Each technique has different advantages and disadvantages and use of complementary approaches may be optimal for the identification of lethal genes relevant for all cancer subtypes. The approach we describe here has a number of advantages over previously developed methods. Firstly, it focuses on specific molecular subtypes and only identifies genes that are expected to be functionally relevant in most/all cases of that particular subtype. This is unlike methods based on identifying partners for specific mutations, which may only be present in a subset of cases and for which different mutations of the same gene could have different lethal partner genes. Unlike the experimental screening approaches, which are often carried out in model systems, our approach identifies selective events occurring in vivo in patients and thus the identified targets will not be model dependent. A limitation of our approach is that it is only able to identify SSV genes that are susceptible to methylation during tumour development and thus won’t detect all potential SSV genes. Consistent with this, we failed to detect SSV gene candidates in one of the nine molecular subtypes analysed in ALL and medulloblastoma, although candidates were identified in the other eight subtypes. Importantly, our approach has a high signal-to-noise ratio, demonstrated by the high hit-rate for validation of SSV genes in the functional studies. While the sensitivity of our approach may be lower than some previously reported methods, the results suggest that the specificity of identification of candidate SSV genes is high. Coupled with the comparative technical simplicity and low costs of the method, we believe that our approach has the potential to significantly enhance the detection of in vivo relevant cancer = -specific lethal genes.

Overall, the approach presented here represents a promising new approach to uncover previously unidentified SSV genes which could represent highly promising targets for the development of treatments with a high-level of cancer specificity and low normal cell toxicity.

## Materials and methods

### Bioinformatic data

For ALL, we used methylation array datasets (GSE49031, GSE69229) [8, 9]. For medulloblastoma, DNA methylation/gene expression for 763 primary medulloblastoma samples was obtained from GSE85218 [33]. All medulloblastoma samples had previously been placed in one of the four consensus medulloblastoma subgroups (WNT, SHH, Group 3 or Group 4) based on integrated analysis of gene expression and genome-wide DNA methylation data [33]. Methylation data were processed as previously described [8]. In total, we used data from 509 samples from seven different leukaemia subtypes (Supplementary Table 2). Analysis of gene expression in ALL was performed using microarray data available from the MILE study (GSE13204; *n* = 637 samples) [15].

### Bioinformatic and statistical analysis

Bioinformatic analyses were undertaken using R v3.4.0 (https://www.r-project.org/foundation). To identify candidate SSV genes, we undertook pairwise comparisons of cancer subtypes. We first identified DMR candidates using DMRcate [34]. DMRs were selected on the basis of average beta-value difference across the full DMR exceeding 0.2. This initial pass was performed by comparing the subtype of interest to all other subtypes combined. From these DMRs, we selected regions for further analysis if the maximum beta-value change at a single locus within the DMR exceeded 0.35 and if the DMR was located within/overlapped a CpG island. For this analysis, the subtype of interest was compared to all other subtypes in pairwise comparisons to ensure that differential methylation was specific for only that subtype. Regions returned by DMRcate are typically variable and contain regions of minimal methylation change as well as regions of large methylation differences. In order to identify the maximally changed regions, we initially selected a minimum of two adjacent CpG loci whose average absolute difference between groups was the greatest. Next, we sought to expand this region by iteratively moving up/downstream of the selected CpGs. We balanced expanding the maximally changed region and maintaining a large absolute methylation change, by incorporating adjacent CpGs into the maximally changed region if the combined average was greater than/equal to the original CpG locus pair difference. We removed DMRs located further than 20 kb from the transcriptional start site of a gene. Using transcriptome microarray data, we identified subtype-specific differentially expressed genes using limma and assessed the expression of candidate SSV genes identified from the DNA methylation screen.

Statistical assessments of differences in cell growth and apoptosis were carried out using the student *t*-test, assuming equal variances, with *p*-values < 0.05 deemed statistically significant.

## Supplementary information

Supplementary Figures and Tables

## Data Availability

Code for identification of SSV genes is available on GitHub. Additional methods in online supplementary material.

## References

[1] Kulis M, Esteller M (2010). DNA methylation and cancer. Adv Genet.

[2] Hanahan D, Weinberg RA (2011). Hallmarks of cancer: the next generation. Cell.

[3] Jacinto FV, Esteller M (2007). Mutator pathways unleashed by epigenetic silencing in human cancer. Mutagenesis.

[4] Kondo Y, Issa JP (2010). DNA methylation profiling in cancer. Expert Rev Mol Med.

[5] Tirado-Magallanes R, Rebbani K, Lim R, Pradhan S, Benoukraf T (2017). Whole genome DNA methylation: beyond genes silencing. Oncotarget.

[6] Capper D, Jones DTW, Sill M, Hovestadt V, Schrimpf D, Sturm D (2018). DNA methylation-based classification of central nervous system tumours. Nature.

[7] Costello JF, Fruhwald MC, Smiraglia DJ, Rush LJ, Robertson GP, Gao X (2000). Aberrant CpG-island methylation has non-random and tumour-type-specific patterns. Nat Genet.

[8] Gabriel AS, Lafta FM, Schwalbe EC, Nakjang S, Cockell SJ, Iliasova A (2015). Epigenetic landscape correlates with genetic subtype but does not predict outcome in childhood acute lymphoblastic leukemia. Epigenetics.

[9] Nordlund J, Backlin CL, Wahlberg P, Busche S, Berglund EC, Eloranta ML (2013). Genome-wide signatures of differential DNA methylation in pediatric acute lymphoblastic leukemia. Genome Biol.

[10] Schwalbe EC, Lindsey JC, Nakjang S, Crosier S, Smith AJ, Hicks D (2017). Novel molecular subgroups for clinical classification and outcome prediction in childhood medulloblastoma: a cohort study. lancet Oncol.

[11] Ross ME, Zhou X, Song G, Shurtleff SA, Girtman K, Williams WK (2003). Classification of pediatric acute lymphoblastic leukemia by gene expression profiling. Blood.

[12] O’Neil NJ, Bailey ML, Hieter P (2017). Synthetic lethality and cancer. Nat Rev Genet.

[13] Oakes CC, Seifert M, Assenov Y, Gu L, Przekopowitz M, Ruppert AS (2016). DNA methylation dynamics during B cell maturation underlie a continuum of disease phenotypes in chronic lymphocytic leukemia. Nat Genet.

[14] Kulis M, Merkel A, Heath S, Queiros AC, Schuyler RP, Castellano G (2015). Whole-genome fingerprint of the DNA methylome during human B cell differentiation. Nat Genet.

[15] Haferlach T, Kohlmann A, Wieczorek L, Basso G, Kronnie GT, Bene MC (2010). Clinical utility of microarray-based gene expression profiling in the diagnosis and subclassification of leukemia: report from the International Microarray Innovations in Leukemia Study Group. J Clin Oncol.

[16] Stoskus M, Vaitkeviciene G, Eidukaite A, Griskevicius L (2016). ETV6/RUNX1 transcript is a target of RNA-binding protein IGF2BP1 in t(12;21)(p13;q22)-positive acute lymphoblastic leukemia. Blood Cells Mol Dis.

[17] Stoskus M, Eidukaite A, Griskevicius L (2016). Defining the significance of IGF2BP1 overexpression in t(12;21)(p13;q22)-positive leukemia REH cells. Leuk Res.

[18] Mazieres J, You L, He B, Xu Z, Lee AY, Mikami I (2005). Inhibition of Wnt16 in human acute lymphoblastoid leukemia cells containing the t(1;19) translocation induces apoptosis. Oncogene.

[19] Li Q, Jedlicka A, Ahuja N, Gibbons MC, Baylin SB, Burger PC (1998). Concordant methylation of the ER and N33 genes in glioblastoma multiforme. Oncogene.

[20] Northcott PA, Shih DJ, Remke M, Cho YJ, Kool M, Hawkins C (2012). Rapid, reliable, and reproducible molecular sub-grouping of clinical medulloblastoma samples. Acta Neuropathol.

[21] Schwalbe EC, Williamson D, Lindsey JC, Hamilton D, Ryan SL, Megahed H (2013). DNA methylation profiling of medulloblastoma allows robust subclassification and improved outcome prediction using formalin-fixed biopsies. Acta Neuropathol.

[22] Moorman AV, Ensor HM, Richards SM, Chilton L, Schwab C, Kinsey SE (2010). Prognostic effect of chromosomal abnormalities in childhood B-cell precursor acute lymphoblastic leukaemia: results from the UK Medical Research Council ALL97/99 randomised trial. lancet Oncol.

[23] Golub TR, Slonim DK, Tamayo P, Huard C, Gaasenbeek M, Mesirov JP, et al. Molecular classification of cancer: class discovery and class prediction by gene expression monitoring. Science. 1999;286:531–7. 10.1126/science.286.5439.531 10521349

[24] Flora A, Klisch TJ, Schuster G, Zoghbi HY. Deletion of Atoh1 disrupts Sonic Hedgehog signaling in the developing cerebellum and prevents medulloblastoma. Science. 2009;326:1424–7. 10.1126/science.1181453 PMC3638077 19965762

[25] Harrison CJ (2011). Acute lymphoblastic leukemia. Clin Lab Med.

[26] Sharma T, Schwalbe EC, Williamson D, Sill M, Hovestadt V, Mynarek M (2019). Second-generation molecular subgrouping of medulloblastoma: an international meta-analysis of Group 3 and Group 4 subtypes. Acta Neuropathologica.

[27] Gao S, Lai L (2018). Synthetic lethality in drug development: the dawn is coming. Future Med Chem.

[28] Thompson JM, Nguyen QH, Singh M, Razorenova OV (2015). Approaches to identifying synthetic lethal interactions in cancer. Yale J Biol Med.

[29] Liu C, Zhao J, Lu W, Dai Y, Hockings J, Zhou Y (2020). Individualized genetic network analysis reveals new therapeutic vulnerabilities in 6,700 cancer genomes. PLoS Comput Biol.

[30] Ryan CJ, Lord CJ, Ashworth A (2014). DAISY: picking synthetic lethals from cancer genomes. Cancer Cell.

[31] Wen YQ, Wu LL, Yang XX, BW Yan, He S, Bo XC. Synthetic lethal interactions prediction based on multiple similarity measures fusion. *Biorxiv* 2020:235366.

[32] Guo J, Liu H, Zheng J (2016). SynLethDB: synthetic lethality database toward discovery of selective and sensitive anticancer drug targets. Nucleic Acids Res.

[33] Cavalli FMG, Remke M, Rampasek L, Peacock J, Shih DJH, Luu B (2017). Intertumoral heterogeneity within Medulloblastoma subgroups. Cancer Cell.

[34] Peters TJ, Buckley MJ, Statham AL, Pidsley R, Samaras K, Lord RV (2015). De novo identification of differentially methylated regions in the human genome. Epigenet Chromatin.

