# Supplementary material for: Integration of genome-level data to allow identification of subtype-specific vulnerability genes as novel therapeutic targets
Source: Oncogene. 2021 Jul 6;40(33):5213–23. doi: 10.1038/s41388-021-01923-1 (PMC8376645; doi:10.1038/s41388-021-01923-1)
Supplement: Supplementary file 1 — Supplementary Figures and Tables [file 41388_2021_1923_MOESM1_ESM.docx]

**Supplementary Figures and Table**

**Fig. S1**

**
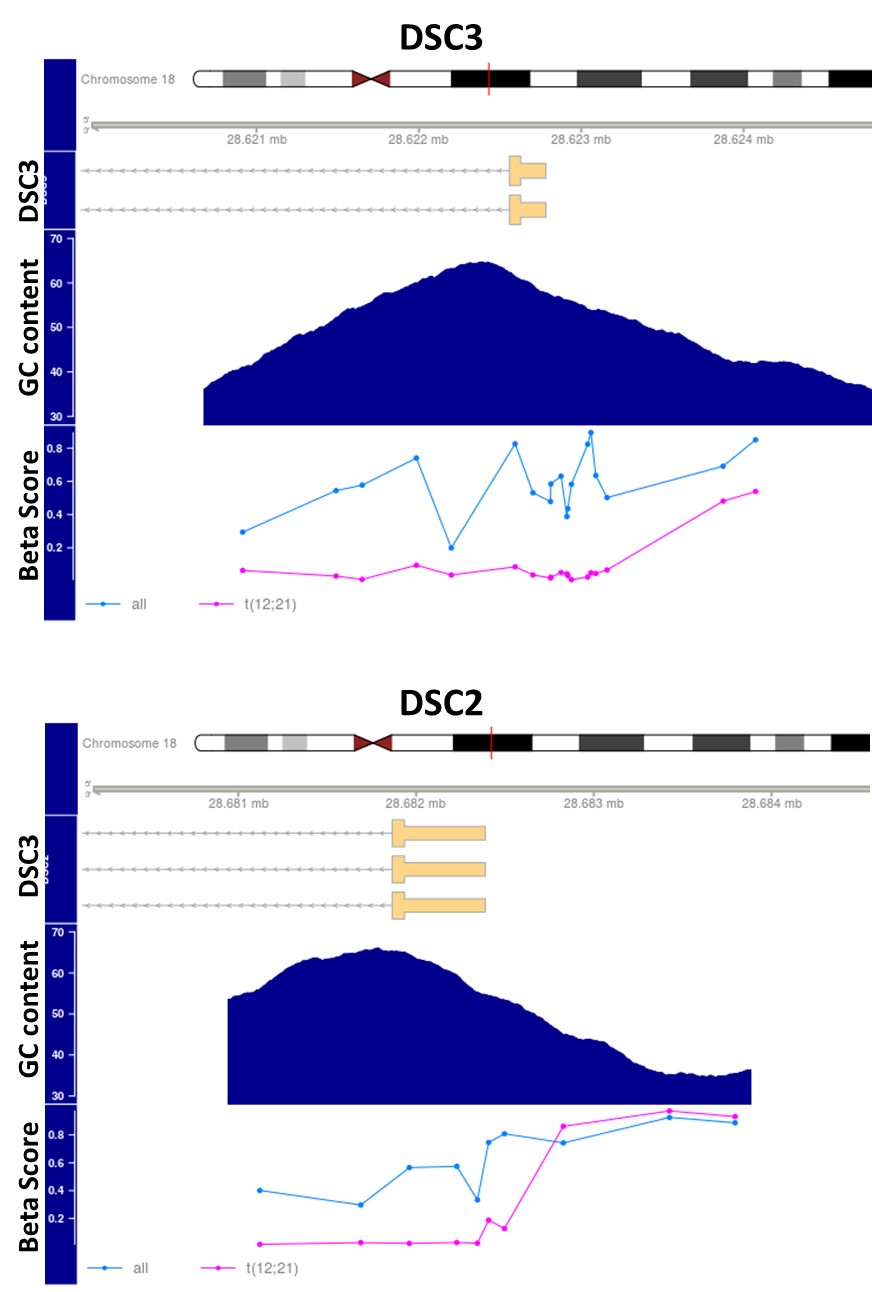
**

**
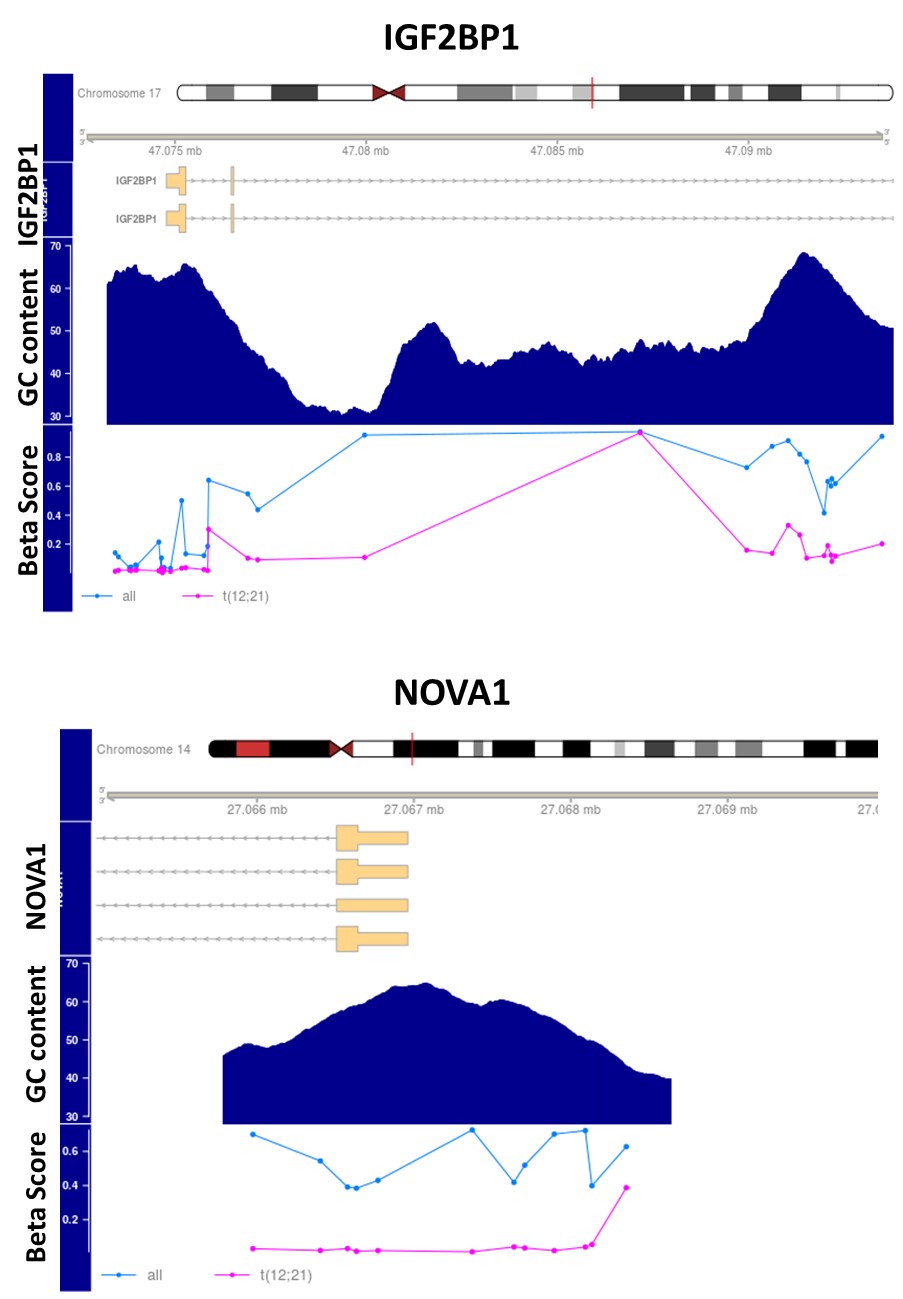
**

**
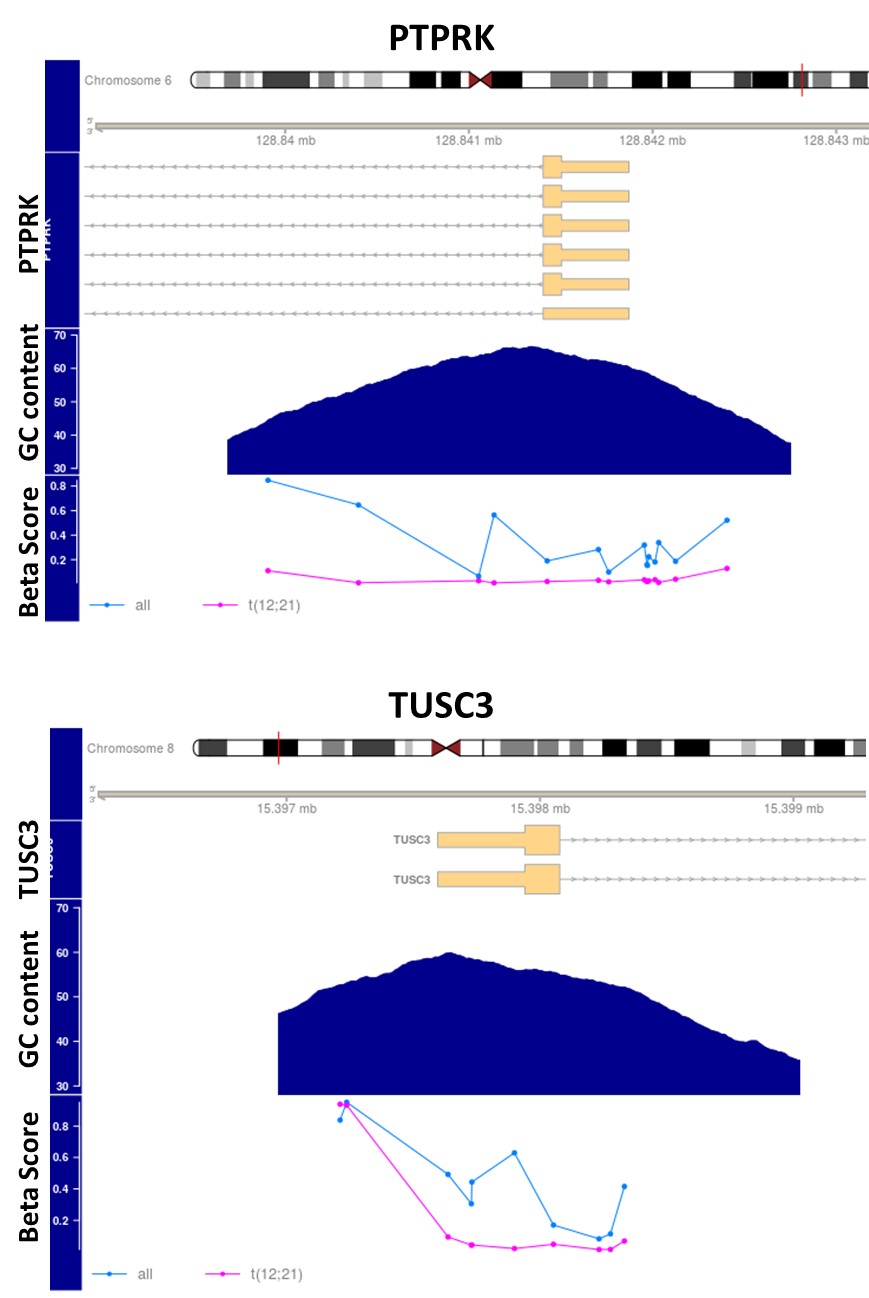
**

**Fig. S1. Genomic context of Identified DMRs in *ETV6-RUNX1* ALL.** DMRs for the 6 candidate SL genes in the *ETV6-RUNX1* subtype are shown relative to the associated gene. Each Figure illustrates the transcriptional start site of the gene, GC content across the region and methylation levels (beta value) at all CpG sites from the DMR that are represented in the 450K-array data. Average methylation levels of *ETV6-RUNX1* cases are in pink and average methylation for all other ALL genetic subtypes is in blue.

**Fig. S2**

**
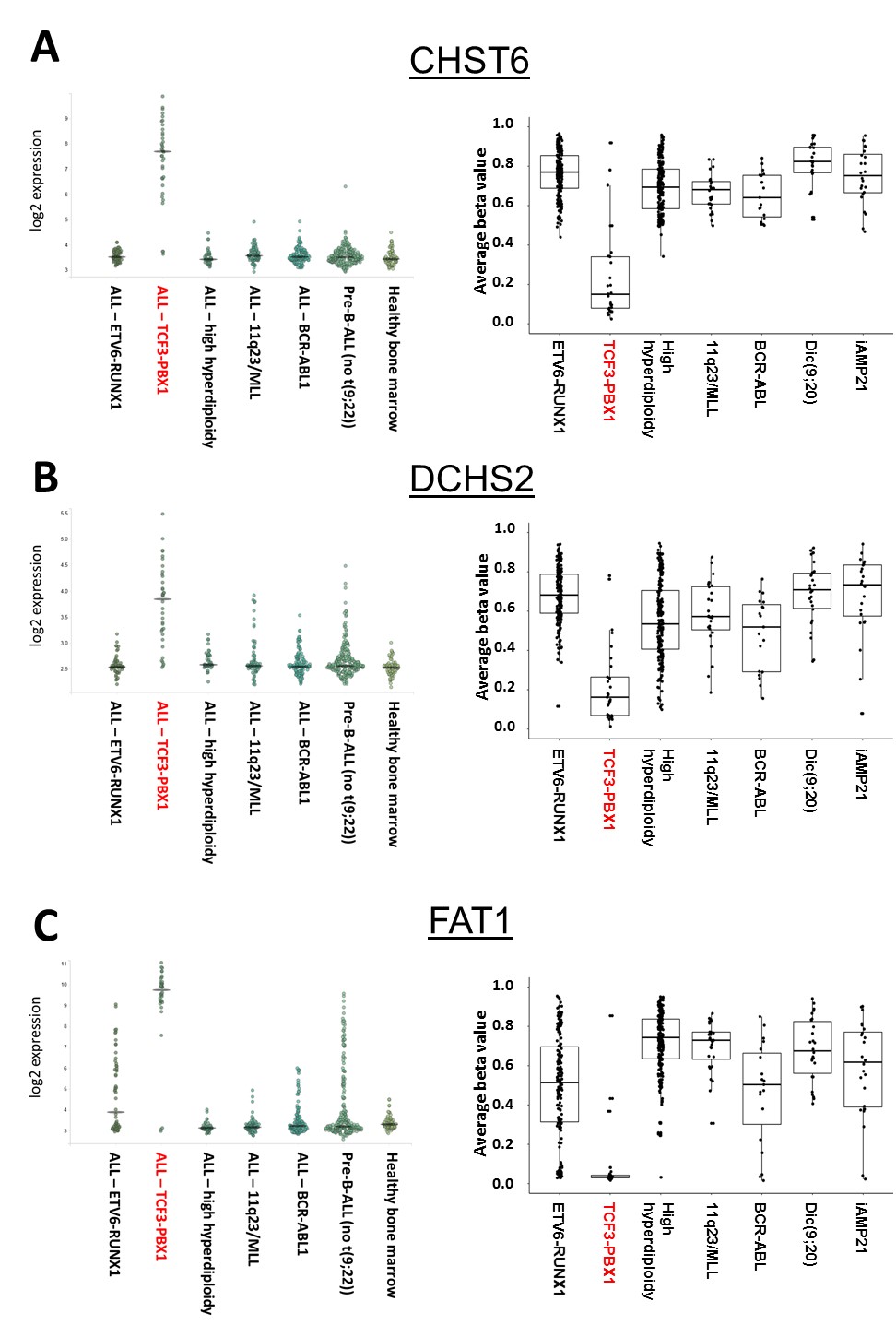
**

**
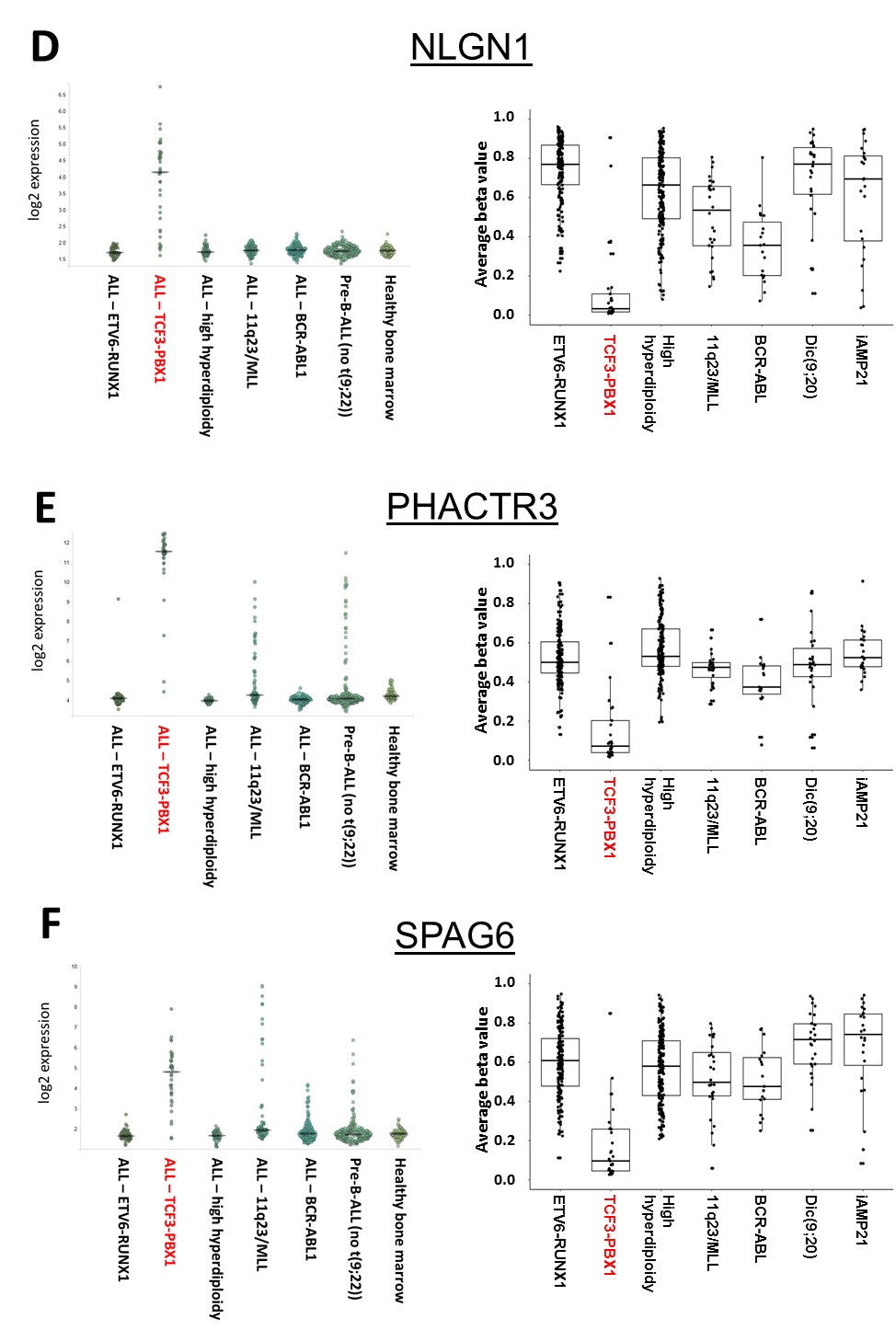
**

**
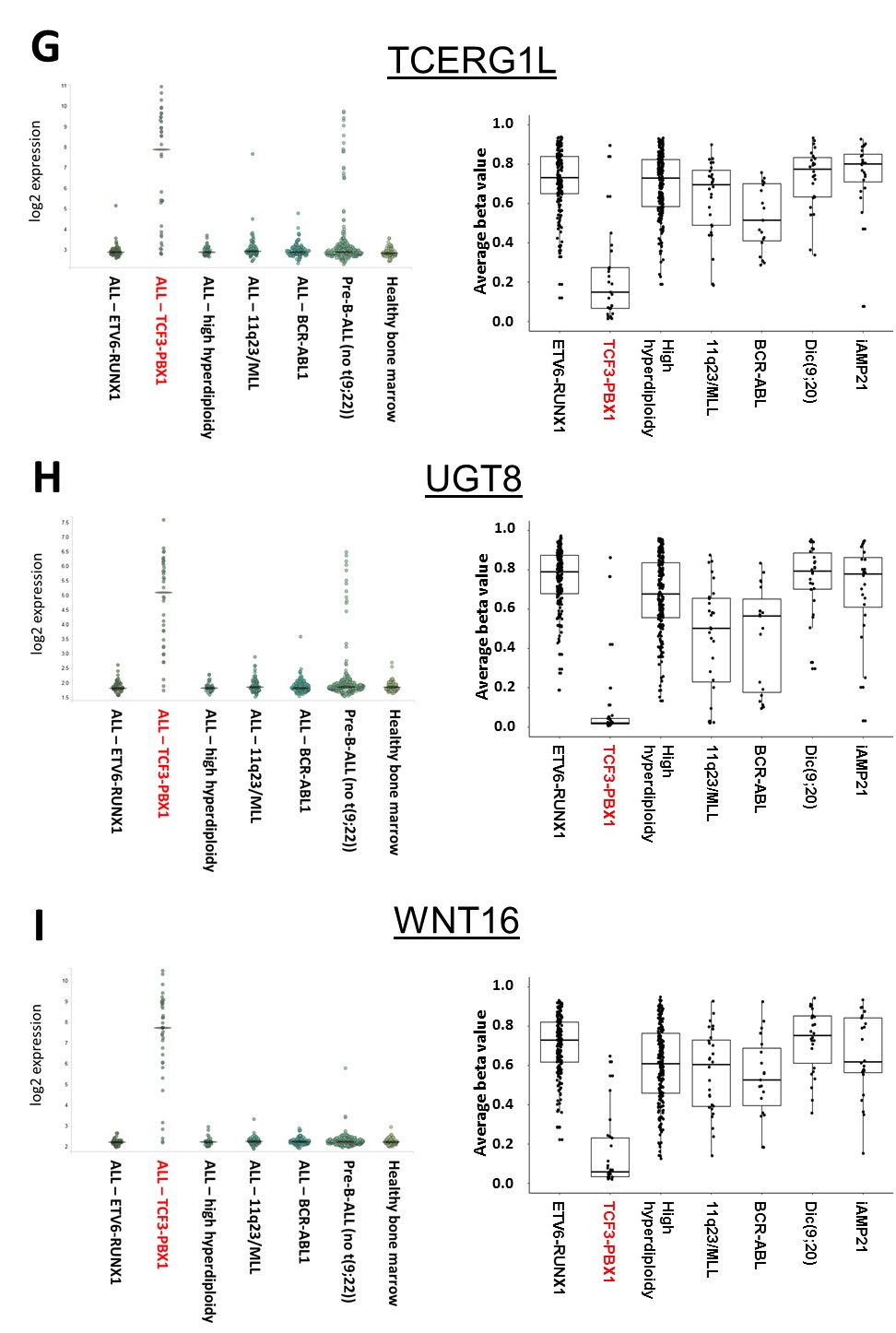
**

**
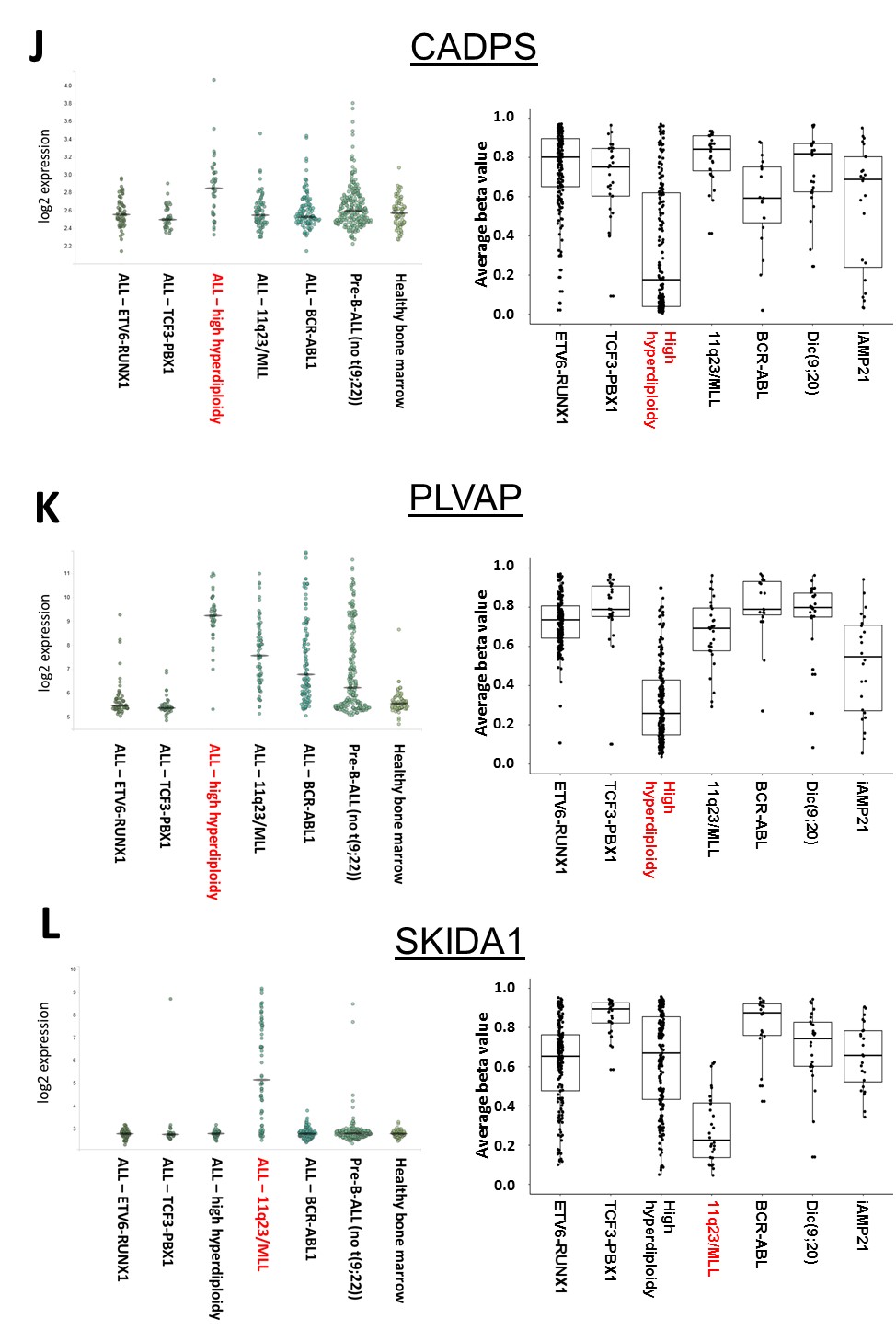
**

**
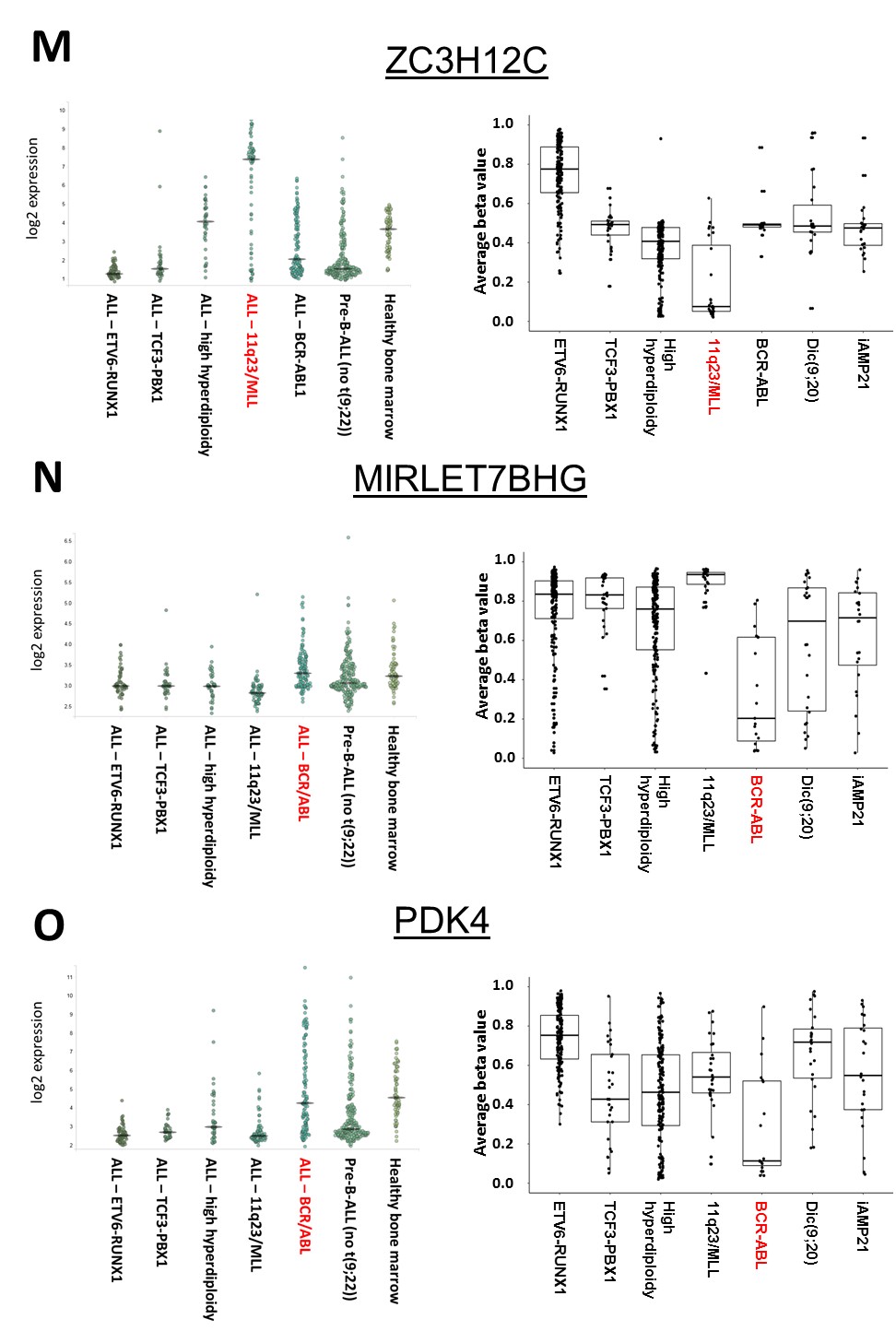
**

**Fig. S2. Methylation and gene expression patterns of SL genes identified in all other ALL genetic subtypes.** Gene expression plots in B-cell ALL genetic subtypes (derived from BloodSpot, <http://servers.binf.ku.dk/bloodspot/>) and DNA methylation data from the region of largest change within each DMR are shown for all candidate SL genes identified in the ALL genetic subtypes. **(A-I)** TCF3-PBX1 SL gene candidates, **(J-K)** High hyperdiploidy SL gene candidates, **(L-M)** 11q23/*MLL* SL gene candidates, **(N-O)** *BCR-ABL* SL gene candidates. For each plot the relevant genetic subtype (in which the gene is predicted to be synthetically lethal) is highlighted in red. The genetic subtypes dic(9;20) and iAMP21 are represented on the methylation plots but not the expression plots as these subtypes were not included in the Leukaemia MILE expression dataset.

**Fig. S3**

**
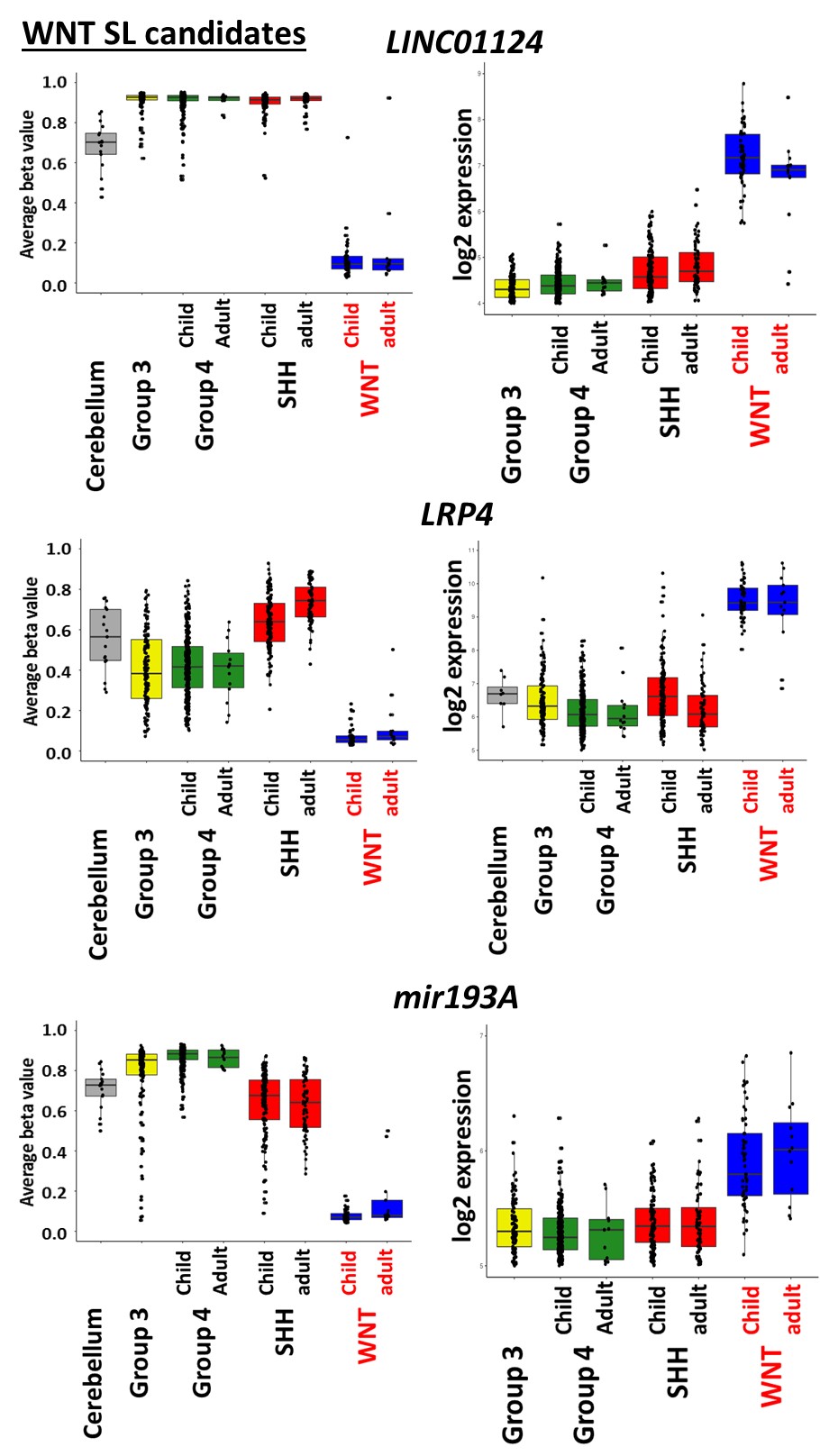
**

**
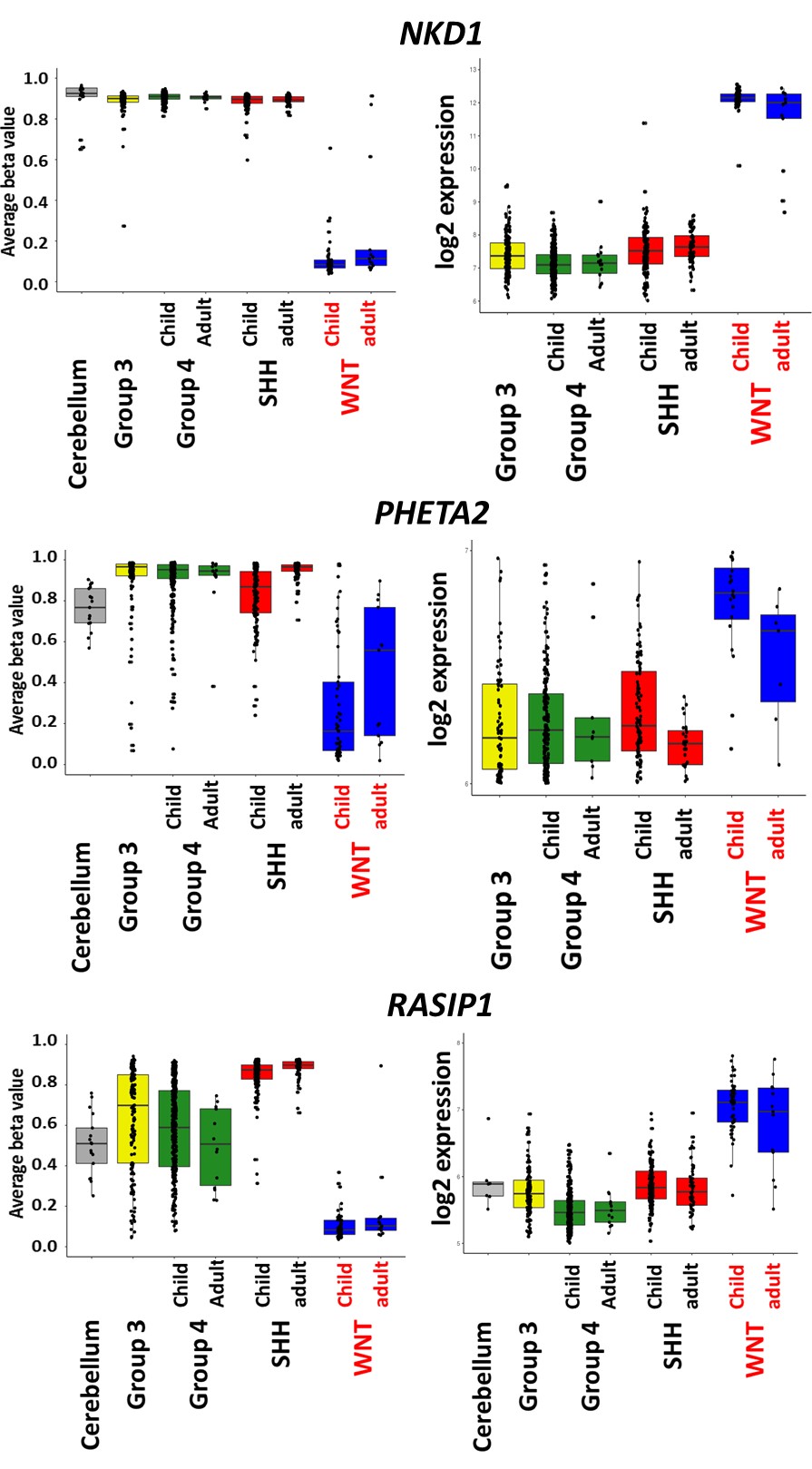
**

**
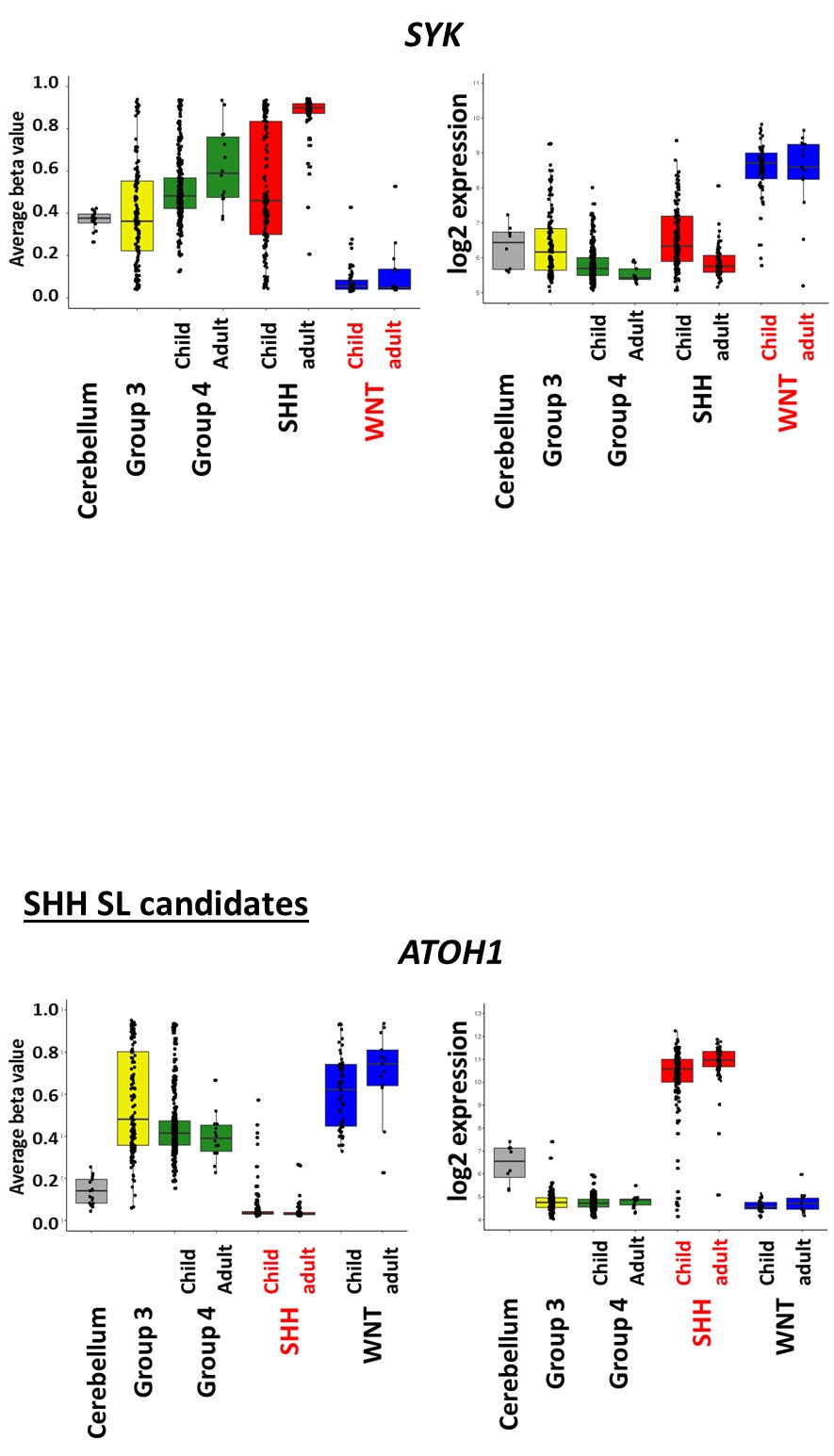
**

**
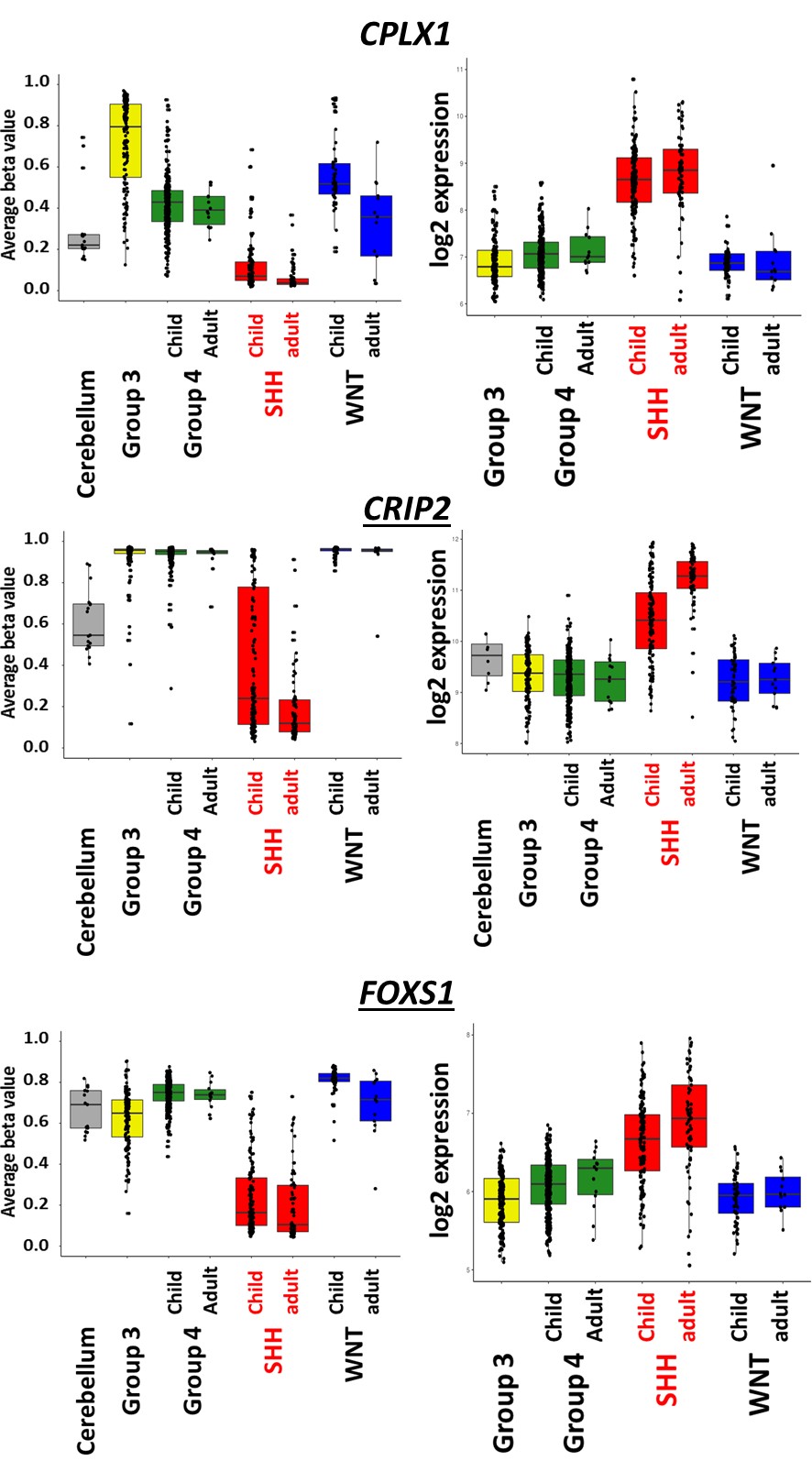
**

**
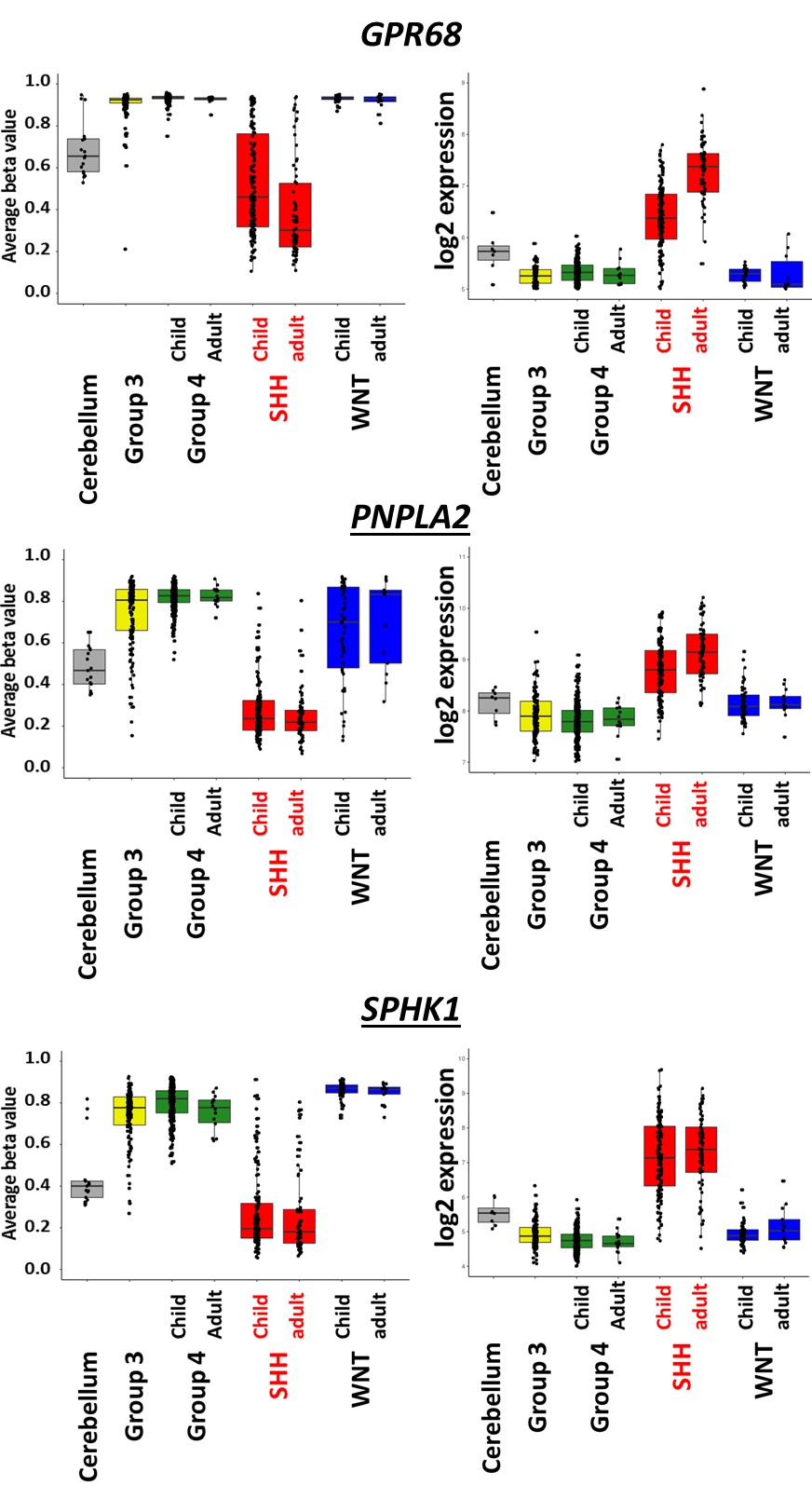
**

**
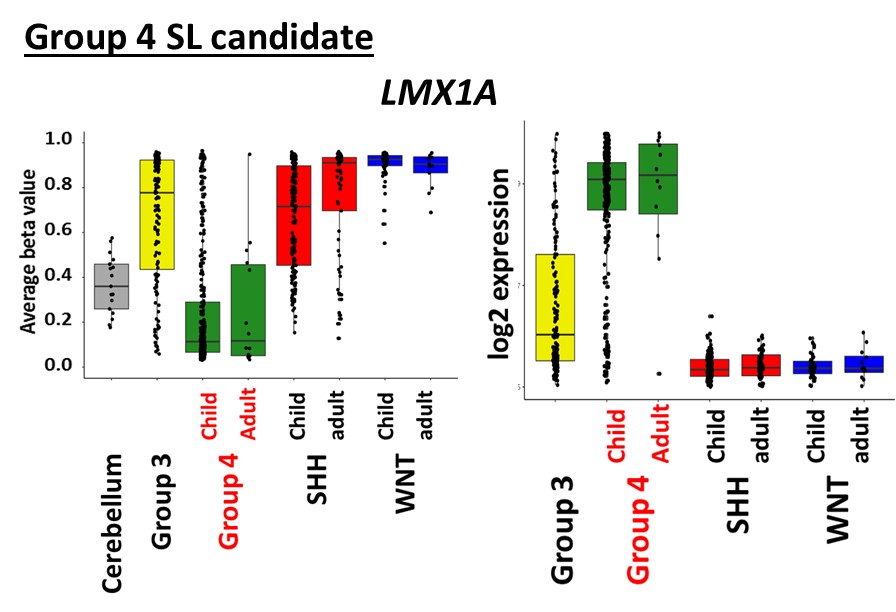
**

**Fig. S3. Methylation and gene expression patterns of SL genes identified in medulloblastoma subtypes.** Gene expression plots DNA methylation data from the region of largest change within each DMR are shown for all candidate SL genes identified in the four medulloblastoma subtypes. For each plot the relevant genetic subtype (in which the gene is predicted to be synthetically lethal) is highlighted in red. For the WNT, SHH and group 4 subtypes the data is shown separately for adult and childhood cases (for group 3 there were not sufficient adult samples to generate meaningful data). Methylation and expression patterns are highly similar in both adult and childhood cases from the same subtype for all 15 identified SL genes, although *PHETA2* (WNT subtype) would not have met the criteria for identification of an SL gene in the adult cases alone.

**Fig. S4**

**A**

**A**


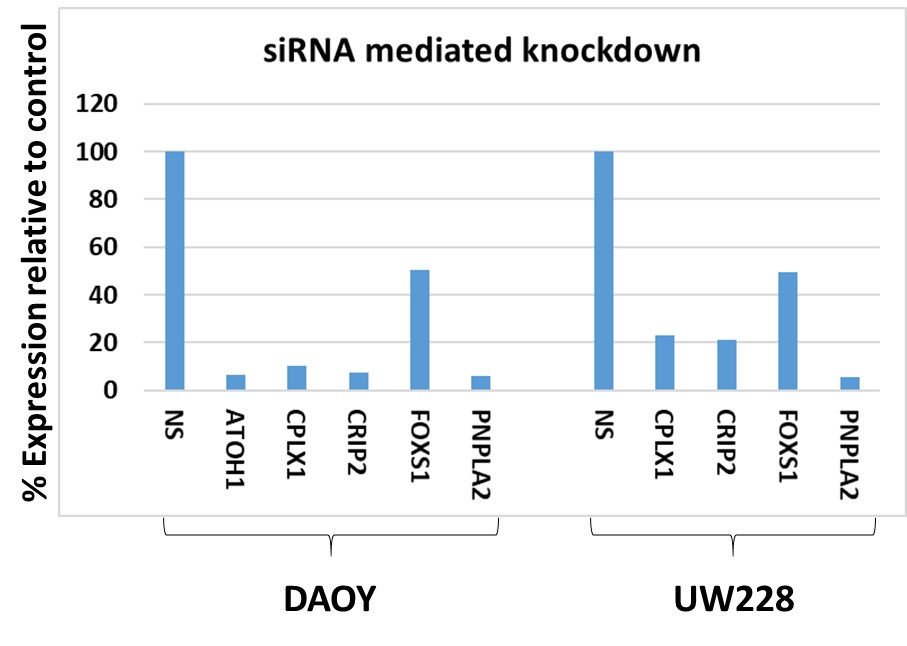


**B**

**
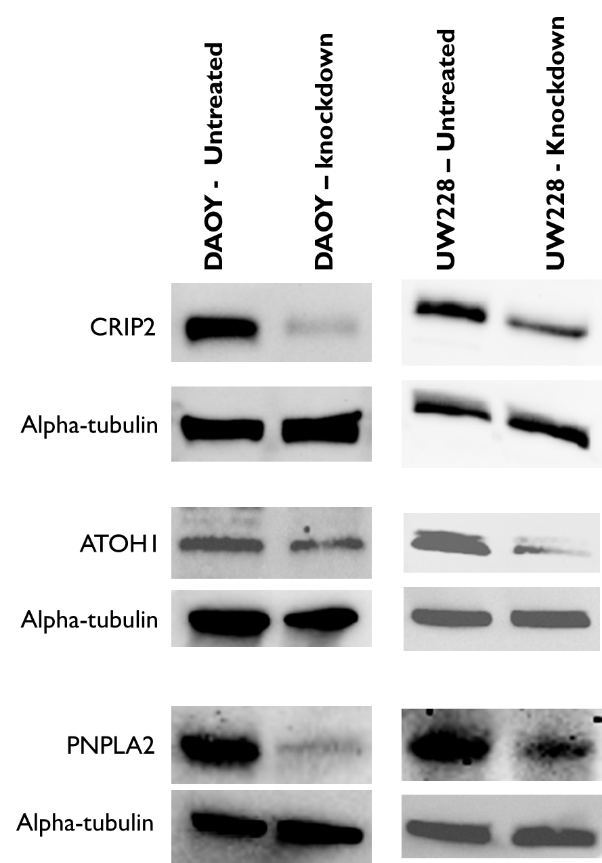
**

**Fig. S4. Transfection with siRNA results in specific repression of the targeted SL gene candidate in medulloblastoma cell lines. A)** RNA expression levels, post transfection, are shown for the five SHH subtype SL candidates that were targeted with siRNA in two SHH subtype medulloblastoma cell lines (DAOY and UW228). Expression was assessed by qRT-PCR 48 hours post transfection. In all cases reductions in expression of between 50 and 95% were observed. For ATOH1 data is only shown for the DAOY cell line. **B)** Protein expression 48hrs post-transfection was assessed after siRNA-mediated knockdown with antibodies specific for CRIP2, ATOH1 and PNPLA2 as indicated. Beta-actin was used as a loading control. For all three genes, protein levels were found to be reduced in both cell lines following siRNA transfection.

**Supplementary Tables**

| **Supplementary Table 1 - Comparison of methylation levels in ALL subtypes and normal cell types for ETV6-RUNX1 related SL genes** | | | | | | | |  |
| --- | --- | --- | --- | --- | --- | --- | --- | --- |
|  |  |  |  |  |  |  |  |  |
| **TUSC3** |  |  |  |  |  |  |  |  |
|  | Methylation differences* | |  | Methylation levels in ALL subtypes and normal cell types^†^ | | | | |
| CpG sites in DMR | Other subtypes vs ETV6-RUNX1 | memCS cells vs naïve cells |  | ETV6-RUNX1 average | Naïve cells average |  | Other ALL subtypes average | memCS cells average |
| cg14202099 | -0.10 | -0.18 |  | 0.90 | 0.79 |  | 0.80 | 0.61 |
| cg19353006 | 0.03 | 0.01 |  | 0.92 | 0.84 |  | 0.95 | 0.85 |
| cg16243756 | 0.33 | 0.21 |  | 0.16 | 0.25 |  | 0.49 | 0.46 |
| cg18145877 | 0.28 | 0.28 |  | 0.09 | 0.07 |  | 0.36 | 0.34 |
| cg03127174 | 0.35 | 0.25 |  | 0.11 | 0.13 |  | 0.45 | 0.39 |
| cg03032098 | 0.49 | 0.41 |  | 0.11 | 0.13 |  | 0.60 | 0.54 |
| cg26374823 | 0.15 | 0.11 |  | 0.07 | 0.16 |  | 0.22 | 0.27 |
| cg07568779 | 0.14 | 0.04 |  | 0.02 | 0.14 |  | 0.17 | 0.08 |
| cg13996963 | 0.16 | 0.15 |  | 0.04 | 0.09 |  | 0.19 | 0.24 |
| cg12089439 | 0.26 | 0.17 |  | 0.15 | 0.23 |  | 0.41 | 0.40 |
|  |  |  |  |  |  |  |  |  |
| **Correlations** |  | r=0.97 |  |  | r=0.99 |  |  | r=0.97 |
|  |  |  |  |  |  |  |  |  |
| **DSC3** |  |  |  |  |  |  |  |  |
|  | Methylation differences | |  | Methylation levels in ALL subtypes and normal cell types | | | | |
| CpG sites in DMR | Other subtypes vs ETV6-RUNX1 | memCS cells vs naïve cells |  | ETV6-RUNX1 average | Naïve cells average |  | Other ALL subtypes average | memCS cells average |
| cg25769889 | 0.19 | 0.03 |  | 0.16 | 0.35 |  | 0.35 | 0.38 |
| cg11814235 | 0.41 | 0.16 |  | 0.13 | 0.23 |  | 0.54 | 0.39 |
| cg25970832 | 0.44 | 0.28 |  | 0.11 | 0.08 |  | 0.56 | 0.36 |
| cg20029745 | 0.51 | 0.31 |  | 0.18 | 0.23 |  | 0.69 | 0.54 |
| cg19967492 | 0.14 | 0.09 |  | 0.10 | 0.15 |  | 0.24 | 0.24 |
| cg15439862 | 0.58 | 0.30 |  | 0.21 | 0.27 |  | 0.79 | 0.58 |
| cg05987251 | 0.38 | 0.22 |  | 0.15 | 0.16 |  | 0.53 | 0.38 |
| cg16492377 | 0.40 | 0.23 |  | 0.10 | 0.12 |  | 0.49 | 0.35 |
| cg11722699 | 0.46 | 0.28 |  | 0.11 | 0.07 |  | 0.57 | 0.35 |
| cg27648075 | 0.48 | 0.32 |  | 0.13 | 0.12 |  | 0.62 | 0.44 |
| cg06263193 | 0.30 | 0.09 |  | 0.11 | 0.04 |  | 0.41 | 0.14 |
| cg26861703 | 0.33 | 0.10 |  | 0.11 | 0.07 |  | 0.45 | 0.18 |
| cg25789861 | 0.45 | 0.19 |  | 0.11 | 0.05 |  | 0.57 | 0.24 |
| cg14631834 | 0.65 | 0.39 |  | 0.14 | 0.16 |  | 0.78 | 0.55 |
| cg11832722 | 0.65 | 0.29 |  | 0.19 | 0.27 |  | 0.84 | 0.56 |
| cg03017520 | 0.45 | 0.23 |  | 0.15 | 0.17 |  | 0.61 | 0.40 |
| cg26497348 | 0.36 | 0.12 |  | 0.13 | 0.30 |  | 0.49 | 0.42 |
| cg14534277 | 0.21 | -0.08 |  | 0.47 | 0.67 |  | 0.68 | 0.59 |
|  |  |  |  |  |  |  |  |  |
| **Correlations** |  | r=0.88 |  |  | r=0.89 |  |  | r=0.79 |
|  |  |  |  |  |  |  |  |  |
| **DSC2** |  |  |  |  |  |  |  |  |
|  | Methylation differences | |  | Methylation levels in ALL subtypes and normal cell types | | | | |
| CpG sites in DMR | Other subtypes vs ETV6-RUNX1 | memCS cells vs naïve cells |  | ETV6-RUNX1 average | Naïve cells average |  | Other ALL subtypes average | memCS cells average |
| cg22017726 | 0.37 | 0.14 |  | 0.06 | 0.08 |  | 0.43 | 0.22 |
| cg06973234 | 0.23 | 0.18 |  | 0.08 | 0.10 |  | 0.32 | 0.29 |
| cg27051931 | 0.41 | 0.33 |  | 0.14 | 0.06 |  | 0.55 | 0.39 |
| cg00196671 | 0.43 | 0.22 |  | 0.13 | 0.12 |  | 0.56 | 0.34 |
| cg00566759 | 0.27 | 0.22 |  | 0.09 | 0.10 |  | 0.37 | 0.32 |
| cg13870990 | 0.45 | 0.19 |  | 0.26 | 0.30 |  | 0.70 | 0.49 |
| cg05110943 | 0.54 | 0.25 |  | 0.22 | 0.32 |  | 0.76 | 0.57 |
| cg05662655 | -0.09 | -0.18 |  | 0.81 | 0.77 |  | 0.73 | 0.60 |
| cg23512701 | -0.09 | -0.06 |  | 0.96 | 0.88 |  | 0.87 | 0.82 |
| cg24505073 | -0.10 | -0.07 |  | 0.93 | 0.82 |  | 0.82 | 0.75 |
|  |  |  |  |  |  |  |  |  |
| **Correlations** |  | r=0.91 |  |  | r=0.99 |  |  | r=0.93 |
|  |  |  |  |  |  |  |  |  |
| **IGFBP1** |  |  |  |  |  |  |  |  |
|  | Methylation differences | |  | Methylation levels in ALL subtypes and normal cell types | | | | |
| CpG sites in DMR | Other subtypes vs ETV6-RUNX1 | memCS cells vs naïve cells |  | ETV6-RUNX1 average | Naïve cells average |  | Other ALL subtypes average | memCS cells average |
| cg21616089 | 0.48 | -0.17 |  | 0.22 | 0.78 |  | 0.70 | 0.61 |
| cg14484274 | 0.59 | -0.18 |  | 0.21 | 0.82 |  | 0.79 | 0.64 |
| cg01885832 | 0.53 | -0.01 |  | 0.37 | 0.77 |  | 0.90 | 0.76 |
| cg20966754 | 0.57 | 0.05 |  | 0.31 | 0.58 |  | 0.78 | 0.64 |
| cg07075026 | 0.54 | 0.12 |  | 0.17 | 0.47 |  | 0.71 | 0.60 |
| cg22947322 | 0.23 | 0.00 |  | 0.14 | 0.42 |  | 0.38 | 0.43 |
| cg10950924 | 0.36 | 0.02 |  | 0.22 | 0.55 |  | 0.58 | 0.56 |
| cg11673840 | 0.43 | 0.03 |  | 0.16 | 0.55 |  | 0.60 | 0.59 |
| cg18128536 | 0.49 | 0.07 |  | 0.11 | 0.54 |  | 0.61 | 0.61 |
| cg25220979 | 0.46 | -0.06 |  | 0.16 | 0.59 |  | 0.62 | 0.53 |
|  |  |  |  |  |  |  |  |  |
| **Correlations** |  | r=-0.12 |  |  | r=0.54 |  |  | r=0.94 |
|  |  |  |  |  |  |  |  |  |
| **NOVA1** |  |  |  |  |  |  |  |  |
|  | Methylation differences | |  | Methylation levels in ALL subtypes and normal cell types | | | | |
| CpG sites in DMR | Other subtypes vs ETV6-RUNX1 | memCS cells vs naïve cells |  | ETV6-RUNX1 average | Naïve cells average |  | Other ALL subtypes average | memCS cells average |
| cg19534149 | -0.04 | -0.11 |  | 0.95 | 0.82 |  | 0.91 | 0.71 |
| cg04850842 | 0.12 | -0.37 |  | 0.26 | 0.68 |  | 0.38 | 0.31 |
| cg23159678 | -0.41 | -0.40 |  | 0.91 | 0.80 |  | 0.50 | 0.40 |
| cg19832521 | 0.51 | 0.26 |  | 0.14 | 0.20 |  | 0.65 | 0.46 |
| cg07559273 | 0.41 | 0.23 |  | 0.11 | 0.06 |  | 0.52 | 0.29 |
| cg20961943 | 0.29 | 0.14 |  | 0.11 | 0.15 |  | 0.40 | 0.29 |
| cg18488855 | 0.32 | 0.22 |  | 0.09 | 0.13 |  | 0.41 | 0.35 |
| cg15602241 | 0.34 | 0.23 |  | 0.09 | 0.10 |  | 0.44 | 0.32 |
| cg20478129 | 0.55 | 0.45 |  | 0.11 | 0.06 |  | 0.66 | 0.51 |
| cg18468511 | 0.32 | 0.25 |  | 0.10 | 0.10 |  | 0.42 | 0.35 |
| cg02014003 | 0.42 | 0.23 |  | 0.10 | 0.19 |  | 0.52 | 0.42 |
| cg07543626 | 0.54 | 0.35 |  | 0.10 | 0.08 |  | 0.64 | 0.43 |
| cg16791424 | 0.60 | 0.28 |  | 0.16 | 0.22 |  | 0.66 | 0.50 |
| cg23502778 | 0.29 | 0.29 |  | 0.15 | 0.11 |  | 0.44 | 0.40 |
| cg11146971 | 0.17 | 0.20 |  | 0.42 | 0.32 |  | 0.59 | 0.53 |
|  |  |  |  |  |  |  |  |  |
| **Correlations** |  | r=0.86 |  |  | r=0.89 |  |  | r=0.91 |
|  |  |  |  |  |  |  |  |  |
| **PTPRK** |  |  |  |  |  |  |  |  |
|  | Methylation differences | |  | Methylation levels in ALL subtypes and normal cell types | | | | |
| CpG sites in DMR | Other subtypes vs ETV6-RUNX1 | memCS cells vs naïve cells |  | ETV6-RUNX1 average | Naïve cells average |  | Other ALL subtypes average | memCS cells average |
| cg20639396 | 0.52 | 0.15 |  | 0.27 | 0.46 |  | 0.79 | 0.62 |
| cg04791477 | 0.41 | 0.22 |  | 0.17 | 0.10 |  | 0.58 | 0.32 |
| cg06726374 | 0.03 | 0.03 |  | 0.07 | 0.12 |  | 0.10 | 0.15 |
| cg22466350 | 0.37 | 0.21 |  | 0.14 | 0.06 |  | 0.51 | 0.27 |
| cg07927488 | 0.15 | 0.10 |  | 0.09 | 0.10 |  | 0.24 | 0.21 |
| cg12148919 | 0.18 | 0.13 |  | 0.14 | 0.13 |  | 0.31 | 0.26 |
| cg25328795 | 0.11 | 0.03 |  | 0.07 | 0.08 |  | 0.18 | 0.11 |
| cg23365490 | 0.21 | 0.04 |  | 0.13 | 0.09 |  | 0.34 | 0.12 |
| cg19033035 | 0.14 | 0.03 |  | 0.10 | 0.05 |  | 0.23 | 0.08 |
| cg15381769 | 0.12 | 0.04 |  | 0.10 | 0.11 |  | 0.22 | 0.15 |
| cg02214443 | 0.18 | 0.06 |  | 0.11 | 0.13 |  | 0.29 | 0.18 |
| cg22179510 | 0.15 | 0.02 |  | 0.10 | 0.07 |  | 0.25 | 0.09 |
| cg02010020 | 0.25 | 0.04 |  | 0.13 | 0.05 |  | 0.37 | 0.10 |
| cg25012434 | 0.17 | 0.03 |  | 0.11 | 0.07 |  | 0.28 | 0.10 |
| cg04023641 | 0.26 | 0.01 |  | 0.23 | 0.44 |  | 0.49 | 0.45 |
|  |  |  |  |  |  |  |  |  |
| **Correlations** |  | r=0.72 |  |  | r=0.84 |  |  | r=0.85 |
|  |  |  |  |  |  |  |  |  |
| *Differences in methylation beta values at individual CpG sites within the identified DMRs are shown for comparison of ETV6-RUNX1 vs all other subgroups (grouped together) and also for comparison of the same CpG sites in naïve B cells vs class-switched memory B cells | | | | | | | | |
|  |  |  |  |  |  |  |  |  |
|  |  |  |  |  |  |  |  |  |
| ^†^Average beta values are shown for individual CpG sites within the identified DMRs for ETV6-RUNX1 ALL/Naïve B cells and then for all other ALL subtypes/class switch memory cells. This shows the very high similarity of methylation patterns between ETV6-RUNX1 ALL/Naïve B cells and between all other ALL subtypes/class switch memory cells at DMRs associated with ETV6-RUNX1 specific SL genes | | | | | | | | |
|  |  |  |  |  |  |  |  |  |
|  |  |  |  |  |  |  |  |  |
|  |  |  |  |  |  |  |  |  |

| **Supplementary Table 2 - Genetic subtypes for which 450K methylation data was available** | | | |
| --- | --- | --- | --- |
|  |  |  |  |
| Genetic subtype | Number of samples |  |  |
| MLL/11q23 | 28 |  |  |
| dic(9;20) | 20 |  |  |
| HeH | 207 |  |  |
| TCF3-PBX1 | 23 |  |  |
| ETV6-RUNX1 | 183 |  |  |
| BCR-ABL1 | 19 |  |  |
| iAMP21 | 29 |  |  |

**Supplementary methods**

**Cell culture**

Leukaemia cell lines were cultured in RPMI 1640 media with L-glutamine and sodium bicarbonate (Sigma-Aldrich, UK); 293T cells and the medulloblastoma cell lines DAOY and UW228 were cultured in Dulbecco’s Modified Eagle’s Medium with 4500mg/l glucose, L-glutamine supplemented with foetal calf serum (Gibco, UK). Cells grown in an incubator at 37⁰C and 5% CO2.

**siRNA transfection**

Cells were re-suspended at 1x10^7^ cells/ml. 200µl (2x10^6^ cells) was added to a 4mm gap electroporation cuvette (VWR, Taiwan). The appropriate volume of siRNA at 20µM/µl (TUSC3 siRNA, non-silencing control (QIAGEN, Netherlands), *FAT1* siRNA (Horizon Discovery, Colorado, USA)), either gene specific or non-silencing control, was added to cuvettes and the cells were electroporated at 310V for 10ms using a EPI 2500 gene pulser (Fischer, Germany). Cells were incubated at 37^o^C for 15 minutes in their cuvettes, then transferred to six well plate or small flasks (Corning Inc., New York, USA) and cultured as detailed above.

The medulloblastoma cell lines DAOY and UW228 cells were transfected with TransIT-X2 Transfection Reagent (Geneflow Limited, UK). The cells were plated at 4X10^5^/ml. cells in 96-well plates 24 hour prior to transfection and transfection was carried out as per manufacturer's instructions. The cells were transfected with one of five gene specific siRNAs (targeting *ATOH1, FOXS1, CPLX1, CRIP2, PNPLA2*) or with control non-silencing siRNA, all obtained from Horizon Discovery.

**Quantitative RT-PCR**

RNA was extracted from cells 48 hours post-electroporation using a Total RNA Purification Kit (Norgen Biotek Corp., Canada) according to the manufacturer’s protocol. Concentration was assessed using a Nanodrop ND-1000 spectrophotometer (Nanodrop, Delaware, USA). cDNA was synthesised using a High-Capacity cDNA Reverse Transcription Kit (Applied Biosystems, UK) according to the manufacturer’s protocol using 2µg of RNA.

Sample were tested in triplicate in a 384-well plate. Each 10µl sample was made from: 0.5µl primers mix (Eurofins Genomics, Luxembourg) at 300ng/µl, 0.5µl cDNA, 4µl nuclease-free water and 5µl Platinum SYBR Green qPCR SuperMix-UDG with ROX (Invitrogen, UK). A water control, and no RT controls were included. The samples were run on a QuantStudio 7 Flex Real-Time PCR system and using QuantStudio Real-Time PCR software v1.2 (both Applied Biosystems). *TUSC3* and *FAT1* primer sequences were: TUSC3-F 5’-atgttcactgctcttcagcc-3’, R 5’-gcagagttcatgttgagctg--3’, FAT1 F 5’- aaagcctgtctgaagtgcag-3’, R 5’- gcttggcatccaatctgatg-3’ and *GAPDH* was as previously reported [35].

**Apoptosis assays**

For leukaemia cell lines, apoptosis was assessed using a PE Annexin V Apoptosis Detection Kit (BD Biosciences, UK) 48 hours post-electroporation, according to the manufacturer’s instructions. Samples were assessed using the FACSCanto II cytometer and FACSDiva software (both BD Biosciences). To ensure accurate compensation, samples with only one of either PE Annexin V or 7-AAD were run as controls.

For Medulloblastoma cell lines (DAOY, UW228) apoptotic levels were assessed using the Caspase-Glo 3/7 Assay System (Promega, UK), at 48-hours post-transfection with siRNA, according to manufacturer’s protocol. Luminescent reading were taken using FLOUstar Omega (BMG LABTECH).

**Assessment of cell growth**

1x10^5^ cells were plated in a total volume of 2ml media in triplicate in a 12-well plate for each sample. Each individual electroporation condition was also performed in duplicate. Analysis of cell numbers was performed either three or five days after transfection. 5µl of 5% Thiazolyl Blue Tetrazolium Bromide (Sigma, UK) in phosphate buffer saline was added, incubated at 37^o^C for 3 hours covered in aluminium foil. 100µl of 10% SDS was added and readings taken on the following day using FLOUstar Omega (BMG LABTECH) plate reader (OD 570nM). Counts were performed in duplicate to ensure reproducibility.

**Lentiviral transduction**

The pSINE-SIEW vector (gift from Dr Paul Sinclair) was used for lentiviral transductions. This vector allows for high efficiency expression of cloned sequences, in addition to eGFP (expressed from the same transcript, but as a separate protein via an internal ribosome entry site). 2x10^6^ cells were transduced using 500µl of lentivirus, either *TUSC3* expressing or control empty vector (expressing eGFP alone) in a total volume of 1.5ml. Cells were washed in PBS 24 hours after transduction, and the efficiency of transduction was initially measured by assessment of GFP expression by flow cytometry at Day 4.

**Western blot**

Proteins were extracted 48 hours post transfection using sonication after treating with SDS-lysis buffer. The BCA Protein Assay Kit (Pierce) was used to estimate protein concentration. 30µg of proteins were loaded onto the gel (Mini-protein precast genes, Bio-Rad). After electrophoresis, proteins were then transferred onto a Polyvinyldifluoride transfer membrane. Membranes were blocked in 5% dried skimmed milk with 0.1% Tween‐20 (TBST) for 1 hr at room temperature, after which they were probed with primary antibodies overnight at 4°C. Primary antibodies used: Rabbit anti-*CRIP2* (Novus biologicals NBP2-59094, 1:1000) and rabbit anti-*ATOH1* (Novus biologicals NBP1-45693, 1:500). For *PNPLA2*, membrane was blocked with 5% dried skimmed milk, 1% BSA in TBS. Membranes were then incubated with rabbit anti-*PNPLA2* (Novus biologicals NB110-41536SS, 1:1000) 1 hour at room temperature. Membranes were then probed with secondary antibody (anti-rabbit IgG, HRP-linked antibody, Cell Signalling Technology, #7074) 1 hour at room temperature. Mouse Anti-α-tubulin (Sigma-Aldrich, T90296, 1:5000) was used for loading control. Blots were developed using SignalFire^TM^ Plus ECL Reagent (Cell Signalling Technology, 12630S) and ChemiDoc^TM^ MP imaging System (Bio-Rad).

Reference

35 Strathdee G, Holyoake TL, Sim A, Parker A, Oscier DG, Melo JV *et al*. Inactivation of HOXA genes by hypermethylation in myeloid and lymphoid malignancy is frequent and associated with poor prognosis. *Clin Cancer Res* 2007; 13: 5048-5055.
